# Proposal of a novel cardiovascular risk prediction score in lupus nephritis

**DOI:** 10.3389/fimmu.2024.1405463

**Published:** 2024-07-24

**Authors:** Adél Molnár, Márk Juha, Klaudia Bulajcsík, Ádám Gy. Tabák, András Tislér, Nóra Ledó

**Affiliations:** ^1^ Department of Internal Medicine and Oncology, Semmelweis University, Budapest, Hungary; ^2^ Institute of Preventive Medicine and Public Health, Semmelweis University Faculty of Medicine, Budapest, Hungary; ^3^ UCL Brain Sciences, University College London, London, United Kingdom

**Keywords:** kidney biopsy, cardiovascular risk, lupus nephritis, prediction tool, major adverse cardiovascular event, systemic lupus erythematosus

## Abstract

**Introduction:**

Patients with systemic lupus erythematosus are prone to develop cardiovascular disease (CVD), and have increased morbidity and mortality.

**Methods:**

We conducted a retrospective analysis on lupus nephritis patients to assess the occurrence and predictors of major adverse cardiovascular events (MACE). Data were collected from patients who underwent kidney biopsy between 2005 and 2020. Statistical analysis was performed to unveil correlations.

**Results:**

91 patients were analyzed in this period, with a mean age of 37.3 ± 12.3 years and 86% being female. The mean follow-up time was 62 ± 48 months. 15.38% of the patients underwent at least one MACE. Two patients deceased of CVD. Increased age (35.81 ± 11.14 vs 45.5 ± 15.11 years, p=0.012) entailed a higher occurrence of MACEs. Neutrophil count (5.15 ± 2.83 vs 7.3 ± 2.99 Giga/L, p=0.001) was higher, whereas diastolic blood pressure (DBP) was lower (89.51 ± 10.96 vs 78.43 ± 6.9 mmHg, p<0.001) at the time of the biopsy in patients with MACE. Age, neutrophil count, and DBP proved to be independent predictors of MACEs. We propose a new model (CANDE – Cardiovascular risk based on Age, Neutrophil count, and Diastolic blood pressure Estimation score) calculated from these variables, which predicts the probability of MACE occurrence.

**Conclusion:**

This study underscores the importance of actively screening for cardiovascular risks in this vulnerable patient population. Age, neutrophil count, and diastolic blood pressure have been established as independent risk factors for MACE in lupus nephritis. The CANDE score derived from these parameters may serve as a prompt, cost-effective, and easily accessible estimation tool for assessing the likelihood of major adverse cardiovascular risk. These findings emphasize the necessity for comprehensive management strategies addressing both immune dysregulation and cardiovascular risk factors in systemic lupus erythematosus to mitigate adverse outcomes.

## 1 Introduction

Systemic lupus erythematosus (SLE) is an autoimmune disorder characterized by multiorgan involvement due to the dysregulation of both the innate and the adaptive immune systems. Renal involvement occurs in 30-50% and contributes vastly to mortality and morbidity. Cardiovascular (CV) risk increases dramatically in SLE and especially in patients with lupus nephritis. It is partly due to the increased level of inflammatory mediators and the accelerated progression of the traditional Framingham risk factors as well as the side effects of the immunosuppressive therapy (1). However, even with the elimination of the traditional risk factors, a drastic excess cardiovascular risk remains compared to the general population (2). This directs the attention towards searching for novel and more SLE-specific mechanisms and predictors of cardiovascular (CV) disease risk.

Our study aimed to find variables that would improve our understanding of the cardiovascular morbidity of patients with lupus nephritis. In this paper, we describe a novel risk prediction model that can be calculated at the time of the kidney biopsy for major cardiovascular events in lupus nephritis.

## 2 Materials and methods

The study cohort comprised Caucasian individuals aged 18 years and above who underwent renal biopsy between 2005 and 2020 at a tertiary-care hospital in the Department of Internal Medicine and Oncology, Semmelweis University, Hungary. The histological processing of specimens was conducted in the Department of Pathology, Forensic and Insurance Medicine, Semmelweis University. The diagnosis of SLE was established based on the 2019 European League Against Rheumatism/American College of Rheumatology (EULAR/ACR) after 2019, the 2012 Criteria for Systemic Lupus Erythematosus (SLICC) criteria between 2012 and 2019, and on the 1997 American College of Rheumatology (ACR) between 1997 and 2012 (3–5).

We collected comprehensive clinical data of the patients retrospectively. Patients underwent a physical examination and blood pressure determination. Our evaluation extended to the participants’ laboratory parameters, medication regimens, age, duration since the diagnosis of lupus, concurrent comorbidities, CV disease history, echocardiographic and electrocardiogram values, and smoking history.

The laboratory tests for immune serology were taken a maximum of three months preceding the biopsy, and regular laboratory parameters were assessed at the time of the biopsy. Immunosuppressive therapy was evaluated at the time of the biopsy and throughout the induction and maintenance phases. Cardiovascular medication use was registered contemporaneously with the biopsy. Blood pressure measurement was not standardized; it was performed using various automatic blood pressure monitors during hospital admission for the biopsy. Cigarette smoking status was determined through self-report.

Major adverse CV events (MACE) were defined as the composite of nonfatal myocardial infarction, hospitalization due to heart failure, coronary revascularization, stroke, and cardiovascular death. We evaluated MACE in the medical history from the time of the diagnosis of lupus and from the time of the biopsy.

Data were stored anonymized in an Excel (Microsoft, version 2016) file. Statistical analysis was conducted with IBM SPSS Statistics v28 software. Figures were formulated in GraphPad Prism 9.0.0 and IBM SPSS Statistics. To compare variables by MACE status, we used Chi-square tests or Fisher’s exact tests for categorical ones, and Mann-Whitney U-tests for continuous ones. To select variables for the multiple logistic regression, we built individual logistic regression models with each predictor that showed significant differences in univariate comparisons as independent and MACE as dependent variable. Given the relatively low number of participants in our dataset, we used these models to select one variable among interrelated factors for the final model based on the fit of the univariate model and the number of data points included. For the final multivariable prediction model, we included all variables selected using the above-described procedure, and then removed non-significant predictors one-by-one in a stepwise manner. For the final multivariable model, Receiver Operating Characteristic (ROC) curve was plotted, and area under the curve (AUC) was calculated to evaluate the model discrimination. Based on this model, we also developed a risk score using a previously established method to predict cardiovascular events in patients with lupus nephritis (6). Two-tailed *p* values <0.05 were considered statistically significant.

The study was conducted in accordance with the Declaration of Helsinki and approved by the Regional and Institutional Committee of Science and Research Ethics of Semmelweis University, Budapest, Hungary (SE RKEB 225/2018). All analyses were performed in accordance with relevant guidelines and regulations, and informed consent was obtained from all subjects and/or their legal guardian(s) for further analyses at the time of the biopsies.

## 3 Results

### 3.1 Demographics

Between 2005 and 2020, 91 adult SLE patients underwent kidney biopsies. The male/female ratio was 14.3%/85.7%, with a mean age of 37.3 ± 12.3 years. The youngest patient was 18 years old, and the eldest patient was 74 years old. The mean follow-up time after the biopsy was 62 ± 48 months. 15.38% (14/91) of the patients suffered at least one major adverse cardiovascular event (MACE) following their lupus diagnosis. Of these, 8.79% (8 out of 91) had such events post-renal biopsy. In total, there were 18 events in 14 patients: three coronary revascularizations, five strokes, six hospitalizations due to heart failure, two acute myocardial infarcts, and two cardiovascular deaths (**Table 1**). Five patients suffered more than one MACE.

**Table 1:** Patients’ characteristics.

Parameters	Overall characteristics^1^
Age (years)	37.3 ± 12.3
Sex (female)	85.7% (78/91)
Follow-up (months)	62 ± 48
MACE^2^ in medical history	15.38% (14/91)
MACE after the kidney biopsy	8.79% (8/91)
Coronary revascularization	3.3% (3/91)
Stroke	5.5% (5/91)
Hospitalization due to heart failure	6.6% (6/91)
Acute myocardial infarction	2.2% (2/91)
Cardiovascular death	2.2% (2/91)

^1^Data are presented either as % (number/all patients) or mean ± SD. ^2^MACE, major adverse cardiovascular event.

### 3.2 Total major adverse cardiovascular events

**Figure 1:**
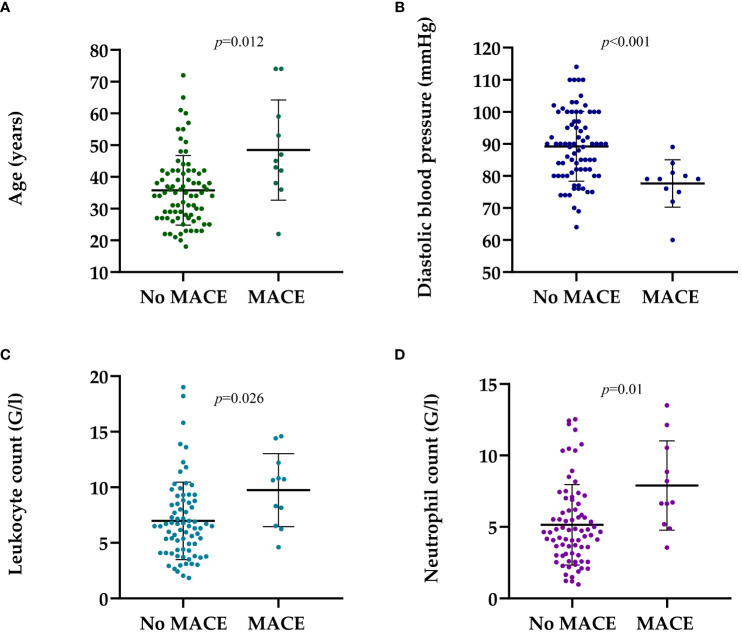
The dot-plots represent the parameters at the time of the kidney biopsy patients who had and had not MACE in medical history. **(A)** Mean (± SD) age of patients with MACE and no MACE was 45.50 ± 15.11 vs. 35.81 ± 11.74 years, p=0.012, respectively. **(B)** Mean (± SD) diastolic blood pressure in patients with MACE and no MACE history was 78.42 ± 6.90 vs. 89.51 ± 10.96 mmHg, p<0.001, respectively. **(C)** Mean (± SD) leukocyte count in patients with MACE and no MACE was 9.07 ± 3.25 vs. 6.99 ± 3.54 G/l, p=0.026, respectively. **(D)** Mean (± SD) neutrophil count in patients with MACE and no MACE was 7.30 ± 3.11 vs. 5.15 ± 2.85 G/L, p=0.01, respectively. Mann-Whitney U-test was used to analyze the differences between the groups. MACE, major adverse cardiovascular event; G/l, Giga/liter; mmHg, Millimeters of Mercury.

Patients with major adverse cardiovascular events were older (45.50 vs 35.81 years; *p*=0.012) (**Figure 1A**), had lower diastolic blood pressure (DBP) (78.42 vs 89.51 mmHg; *p*<0.001) (**Figure 1B**), higher leukocyte count (9.07 vs 6.99 Giga/liter; *p*=0.026) (**Figure 1C**), and absolute neutrophil count (7.30 vs 5.15 Giga/liter; *p*=0.01) (**Figure 1D**, **Table 2**, **Supplementary Table S1**). Neither leukocyte count nor the elevation of absolute neutrophil count was associated with steroid administration, or steroid dosage (r=0.097 *p*=0.375; r=0.110 *p*=0.315, respectively).

**Table 2:** Patient characteristics by major adverse cardiovascular event status for variables that have data for at least 75% of participants.

Variables	MACE in the medical history^1^	No MACE in the medical history^1^	*p*
Age (years)	45.50 ± 15.11 (42.50) n=14	35.81 ± 11.74 (35.00) n=77	*0.012*
Sex (females)	92.9% (13/14)	84.4% (65/77)	0.406
Time from lupus diagnosis to biopsy (years)	8.80 ± 10.12 (5.50) n=14	6.84 ± 6.74 (5.00) n=75	0.640
*Clinical and general laboratory parameters*			
Systolic blood pressure (mmHg)	132.50 ± 18.73 (127.50) n=14	141.18 ± 19.92 (140.00) n=74	0.069
Diastolic blood pressure (mmHg)	78.42 ± 6.90 (79.00) n=14	89.51 ± 10.96 (90.00) n=74	*<0.001*
Pulse pressure (mmHg)	54.07 ± 19.33 (48.00) n=14	51.66 ± 13.61 (52.00) n=74	0.842
Leukocyte count (G/l)	9.07 ± 3.25 (8.45) n=14	6.99 ± 3.54 (6.50) n=73	*0.026*
Hemoglobin (g/l)	112.21 ± 18.53 (109.00) n=14	108.52 ± 18.97 (106.00) n=73	0.533
Hematocrit (l/l)	0.34 ± 0.07 (0.34) n=14	0.33 ± 0.06 (0.32) n=73	0.595
Neutrophil (%)	79.14 ± 9.3 (81.25) n=14	72.5 ± 12.06 (73.80) n=73	0.058
Neutrophil count (G/l)	7.30 ± 3.11 (6.68) n=14	5.15 ± 2.85 (4.65) n=73	*0.010*
Lymphocyte (%)	15.01 ± 8.26 (14.05) n=14	19.25 ± 10.06 (17.80) n=73	0.146
Lymphocyte count (G/l)	1.29 ± 0.74 (1.26) n=14	1.30 ± 0.98 (1.00) n=73	0.599
Platelet count (G/l)	270.07 ± 100.70 (290.00) n=14	245.63 ± 101.25 = 242.00) n=73	0.212
Sodium (mmol/l)	139.92 ± 3.25 (140.00) n=13	139.85 ± 3.62 (140.00) n=71	0.955
Potassium (mmol/l)	4.37 ± 0.58 (4.30) n=13	4.36 ± 0.61 (4.30) n=72	0.536
Calcium (mmol/l)	2.16 ± 0.16 (2.12) n=12	2.15 ± 0.22 (2.15) n=63	0.745
Phosphate (mmol/l)	1.38 ± 0.25 (1.35) n=11	1.28 ± 0.34 (1.27) n=60	0.206
Serum albumin (g/l)	30.65 ± 7.78 (27.80) n=11	31.19 ± 6.91 (30.75) n=62	0.717
CRP^2^ (mg/l)	10.86 ± 13.60 (7.85) n=12	7.91 ± 6.91 (3.6) n=62	0.304
GFR^3^ (ml/min/1.43 m^2^)	35.37 ± 14.15 (35.55) n=6	47.77 ± 21.88 (49.60) n=33	0.184
Creatinine (µmol/l)	116.15 ± 110.79 (87.00) n=13	112.33 ± 89.27 (82.50) n=72	0.831
BUN^4^ (mmol/l)	11.98 ± 8.70 (10.30) n=13	10.13 ± 7.45 (7.75) n=72	0.376
Hematuria (erythrocyte/HPF^5^)	123.43 ± 366.56 (5.50) n=14	27.68 ± 50.95 (10.00) n=71	0.648
Leukocyturia (leukocyte/HPF^5^)	14.29 ± 19.41 (11.50) n=14	19.06 ± 46.39 (10.00) n=68	0.951
NLR^6^	7.29 ± 4.99 (5.87) n=14	5.68 ± 4.79 (4.18) n=73	0.101
NPR^7^	0.03 ± 0.02 (0.03) n=14	0.02 ± 0.02 (0.02) n=73	0.111
PLR^8^	288.40 ± 232.37 (177.88) n=14	297.87 ± 315.12 (225.97) n=73	0.881
*Auto-antibodies, lupus-specific laboratory parameters*			
C3 (g/l)	0.69 ± 0.23 (0.67) n=10	0.62 ± 0.29 (0.59) n=60	0.411
C4 (g/l)	0.08 ± 0.04 (0.07) n=10	0.11 ± 0.11 (0.07) n=60	0.880
ANA^9^ positivity	100.0% (13/13)	93.3% (56/60)	0.338
Anti-dsDNA^10^ positivity	63.6% (7/11)	90.5% (57/63)	*0.016*
*Comorbidities*			
Hypertension	42.9% (6/14)	38.2% (29/76)	0.740
Diabetes mellitus	14.3% (2/14)	2.6% (2/76)	0.113
Deep vein thrombosis	50.0% (7/14)	14.3% (11/77)	*0.002*
Antiphospholipid syndrome	35.7% (5/14)	7.8% (6/77)	*0.011*
Pericardial effusion	14.3% (2/14)	11.7% (9/77)	0.534
Smoking	33.3/(4/12)	27.0% (17/63	0.729
*Medication at the time of the kidney biopsy*			
Vitamin D3	21.4% (3/14)	29.9% (23/77)	0.749
Anticoagulant	57.1% (8/14)	19.5% (15/77)	*0.003*
Thrombocyte aggregation inhibitor	21.4% (3/14)	6.5% (5/77)	0.102
Calcium channel blocker	28.6% (4/14)	27.3% (21/77)	0.575
Spironolactone	14.3% (2/14)	2.6% (2/77)	0.110
Furosemide	50.0% (7/14)	32.5% (25/77)	0.206
Thiazide/thiazide-like diuretics	21.4% (3/14)	13.0% (10/77)	0.683
ACE-I/ARB^11^	71.4% (10/14)	44.2% (34/77)	0.060
Statin	28.6% (4/14)	9.1% (7/77)	0.062
Beta blocker	50.0% (7/14)	22.1% (17/77)	*0.029*
Antimalarial medication	7.1% (1/14)	6.8% (5/74)	0.658
Methotrexate	0.0% (0/14)	4.1% (3/74)	0.591
Mycophenolate mofetil	0.0% (0/14)	4.1% (3/74)	0.591
Azathioprin	7.1% (1/14)	10.8% (8/74)	0.563
Cyclosporin A	0.0% (0/14)	4.1% (3/74)	0.591
Cyclophosphamide	0.0% (0/14)	5.4% (4/74)	0.611
Glucocorticoids	92.9% (13/14)	81.1% (60/74)	0.283
*Remission induction therapy*			
EUROLUPUS^12^ induction	54.5% (6/11)	52.9% (37/70)	0.917
Cyclophosphamide induction^13^	0.0% (0/11)	7.1% (5/70)	0.606
Glucocorticoids induction only	0.0% (0/11)	25.7% (18/70)	0.111
Plasmapheresis upon induction	0.0% (0/11)	4.3% (3/70)	0.642
Glucocorticoids and calcineurin inhibitor induction	0.0% (0/11)	1.4% (1/70)	0.864
Mycophenolate mofetil	0.0% (0/11)	15.7% (11/70)	0.345
IVIG^14^ upon induction	0.0% (0/12)	2.8% (2/72)	0.733
*Maintenance therapy*			
Methotrexate	10.0% (1/10)	1.4% (1/69)	0.258
Mycophenolate mofetil	20.0% (2/10)	26.1% (18/69)	0.596
Calcineurin inhibitor maintenance	20.0% (2/10)	8.7% (6/69)	0.591
Antimalarial medication	20.0% (2/10)	21.7% (15/69)	0.873
Azathioprin	30.0% (3/10)	31.9% (22/69)	0.531
Cyclophosphamide	0.0% (0/10)	14.5% (10/69)	0.342
Glucocorticoids	100.0% (10/10)	94.2% (65/69)	0.435
*Remission – relapses*			
Complete remission at 1 year	75.0% (6/8)	53.1% (34/64)	0.240
Partial remission at 1 year	12.5% (1/8)	20.3% (13/64)	0.594
No remission at 1 year	12.5% (1/8)	26.6% (17/64)	0.437
Relapse in 3 years	66.7% (6/9)	65.4% (34/52)	0.940
*Histopathological data*			
Class I	7.1% (1/14)	1.3% (1/77)	0.285
Class II	7.1% (1/14)	5.2% (4/77)	0.575
Class III	14.3% (2/14)	23.4% (18/77)	0.727
Class IV	50.0% (7/14)	55.8% (43/77)	0.774
Class V	28.6% (4/14)	19.5% (15/77)	0.480
Class VI	0.0% (0/14)	2.6% (2/77)	1.000
Overall distribution of the Classes			0.772

^1^Data are presented either as % (number/all patients) or mean ± SD (median) n= number of patients. MACE, major adverse cardiovascular event, ^2^CRP, C-reactive protein, ^3^GFR, Glomerular filtration rate, ^4^BUN, Blood Urea Nitrogen, ^5^HPF, high-power field, ^6^NLR, Neutrophil-Lymphocyte ratio, ^7^NPR, Neutrophil-Platelet ratio, ^8^PLR, Platelet-Lymphocyte ratio, ^9^ANA, anti-nuclear antibodies, ^10^dsDNA, double-stranded deoxyribonucleic acid, ^11^ACE-I/ARB, angiotensin-converting enzyme inhibitor/angiotensin II receptor blocker, ^12^EUROLUPUS, glucocorticoids and cyclophosphamide, ^13^Non-cyclic oral cyclophosphamide, ^14^IVIG, intravenous immunoglobulin. Chi-square analysis or Mann-Whitney U-test were utilized to calculate *p*-values. Significant p-values are in italics.

**Figure 2:**
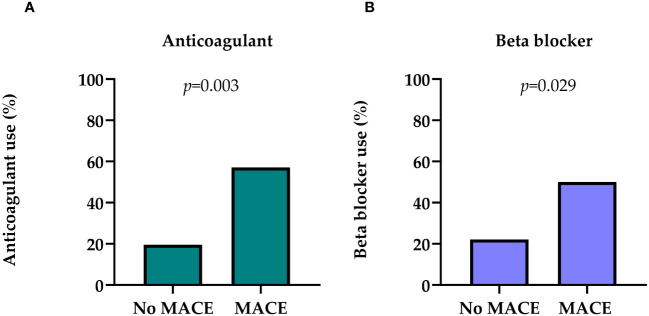
**(A)** Distribution of anticoagulant use in patients with MACE and no MACE in medical history. **(B)** Distribution of beta blocker use in patients with MACE and no MACE in the medical history. Chi square test was used to analyze the differences between the groups. MACE, major adverse cardiovascular event.

Patients with MACE were more often on anticoagulant (57.1% vs. 19.5%; *p*=0.003) (**Figure 2A**), and beta blocker (50.0% vs. 22.1%; *p*=0.029) medication at the time of renal biopsy (**Figure 2B**). Antihypertensive and diuretic medication use had no discernible influence on the on MACE risk (**Supplementary Table S2**).

**Figure 3:**
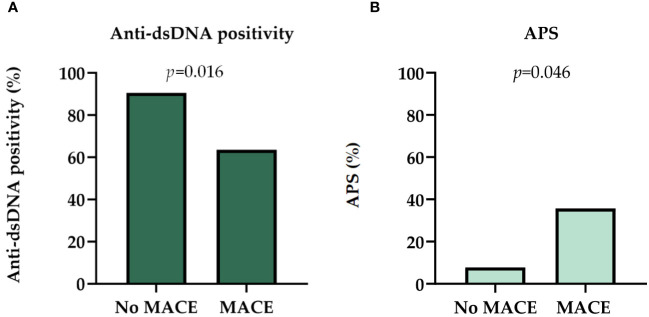
**(A)** Distribution of anti-dsDNA positivity in patients with MACE and no MACE at the time of the biopsy. **(B)** Distribution of antiphospholipid syndrome in patients at the time of the biopsy. Chi square test was used to analyze the differences between the groups. APS, antiphospholipid syndrome; dsDNA, double-stranded deoxyribonucleic acid; MACE, major adverse cardiovascular event.

Patients with MACE less frequently had anti-dsDNA positivity (63.6% vs. 90.5%; *p*=0.016) (**Figure 3A**). Anti-dsDNA positivity was associated with lower absolute neutrophil count (5.08 vs 7.44 Giga/liter, *p*=0.035). Proteinuria did not show statistically significant impact on the occurrence of MACE (*p*=0.359).

Pulse pressure was non-significantly wider in individuals with MACE (54.07 vs 51.66 mmHg; *p*=0.842) (**Table 2**). Antiphospholipid syndrome (APS), as well as deep vein thrombosis in the medical history was more frequently present at the time of kidney biopsy among those with MACE events (35.7% vs. 7.8%; *p*=0.011; 50.0% vs. 14.3%, p=0.02) (**Table 2**, **Supplementary Table S1**, **Figure 3B**). However, it is important to note that all patients with APS were on anticoagulants, but not all anticoagulated patients had APS (**Supplementary Figure S1**).

Our analysis revealed no significant association between the occurrence of MACE and remission status (no remission: *p*=0.953, partial remission: *p*=0.790, 3-year relapse: *p*=0.953).

Univariate logistic regression indicated an association between MACE and older age (OR 1.059 per 1 year, *p*=0.011), lower DBP (OR 0.889 per 1 mmHg, *p*=0.002), higher absolute neutrophil count (OR 1.248 per 1 G/l, *p*=0.018), anticoagulant (OR 6.000, p=0.004) and beta blocker use (OR 3.529, *p*=0.036), absence of anti-dsDNA positivity (OR 0.184, *p*=0.026), presence of antiphospholipid syndrome (OR 6.574, p=0.007), and deep vein thrombosis (OR 6.000, p=0.004) (**Table 3**).

**Table 3:** Risk factors for major adverse cardiovascular events.

Variables	B	*p*	OR^1^	Confidence Interval for OR	
				Lower	Upper
*Univariate Logistic Regression*					
Age (years)	0.057	***0.011***	1.059	1.013	1.017
Diastolic blood pressure (mmHg)	-0.118	***0.002***	0.889	0.824	0.958
Leukocyte count (G/l)	0.148	0.053	1.160	0.998	1.347
Neutrophil count (G/l)	0.222	***0.018***	1.248	1.039	1.499
Anticoagulant	1.792	***0.004***	6.000	1.795	20.052
Beta blocker	1.261	***0.036***	3.529	1.087	11.462
Anti-dsDNA^2^	-1.692	***0.026***	0.184	0.042	0,816
Antiphospholipid syndrome	1.883	***0.007***	6.574	1.663	25.990
Deep vein thrombosis	1.792	***0.004***	6.000	1.759	20.461

^1^OR, odds ratio, ^2^dsDNA, double-stranded deoxyribonucleic acid. Significant *p*-values are in italics and bold.

### 3.3 Subgroup analysis of patients with major adverse cardiovascular event in the medical history

The subgroup analysis was constrained by the modest number of patients; however, the following associations may be noted.

Patients with a history of coronary revascularization demonstrated an elevated neutrophil-platelet ratio (0.06 vs 0.02; *p*=0.02) (**Supplementary Table S3**). Patients with a stroke were older (56.20 vs. 36.20 years; *p*=0.017) and had lower DBP (78.00 vs 88.34 mmHg; *p*=0.018) (**Supplementary Table S4**). Patients with hospitalization due to heart failure were more frequently smokers (78.0% vs 25.4%; *p*=0.031) and had higher C-reactive protein level (18.13 vs 7.52 mg/L; *p*=0.021) (**Supplementary Table S5**). Myocardial infarction occurred in two cases and two patients died of cardiovascular causes in this period (**Supplementary Tables S6**, **S7**).

### 3.4 Assessment of long-term cardiovascular risk in lupus nephritis patients

Among interrelated factors (such as neutrophil count/anti-dsDNA positivity and deep vein thrombosis/antiphospholipid syndrome/anticoagulant use), one variable was selected based on the fit of the univariate model and the number of available data points. For the final multivariable prediction model, we included all variables selected using the above-described procedure (age, DBP, neutrophil count, beta blocker and anticoagulant use). Subsequently, non-significant predictors were systematically removed in a stepwise manner (**Supplementary Figure S2**). Finally, among the variables that showed a significant association with MACE based on univariate logistic regressions, only lower diastolic blood pressure, higher neutrophil count, and older age at the time of the biopsy remained as independent risk factors for MACE (**Table 4**). Using these findings, we devised a scoring system (the CANDE score – Cardiovascular risk-based on Age, Neutrophil count, and Diastolic blood pressure Estimation Score) to assess cardiovascular risk in patients with lupus nephritis. This system is designed to predict the probability of major adverse cardiovascular events (MACE) at the time of renal biopsy.

**Table 4:** Independent risk factors for major adverse cardiovascular events based on multivariable logistic regression that provide the basis of the CANDE (Cardiovascular risk –based on Age, Neutrophil count, and Diastolic blood pressure Estimation) score.

Variables	B	*p*	OR^1^	Confidence Interval for OR	
				Lower	Upper
*Multivariate Logistic Regression*					
Age (years)	0.052	***0.048***	1.053	1.000	1.109
Diastolic blood pressure (mmHg)	-0.124	***0.005***	0.884	0.810	0.964
Neutrophil count (G/l)	0.278	***0.020***	1.320	1.044	1.668
*CANDE^2^ score*					
*MACE^3^ in medical history*	0.128	***<0.001***	1.137	1.062	1.217
*MACE after the kidney biopsy*	0.078	***0.030***	1.081	1.019	1.147

^1^OR, odds ratio, ^2^CANDE, Cardiovascular risk –based on Age, Neutrophil count, and Diastolic blood pressure Estimation Score, ^3^MACE, major adverse cardiovascular event. Significant *p*-values are in italics and bold.

The rounded beta values (coefficients) from the final multivariable logistic regression (**Table 4**) were used as weighting factors for the calculation of the CANDE score. To simplify the calculation, the effect of age was calculated for 10-year intervals. The CANDE-score is a linear combination of the values of the features and their corresponding weights. By applying logistic regression with the CANDE score as the independent variable, we demonstrated that a 1-point higher CANDE score is associated with a 13.7% increase of MACE risk (*p*<0.001) (**Table 4**). The score has showed good calibration (Hosmer-Lemeshow test X^2^ = 2.322, *p*=0.970).

**Figure 4:**
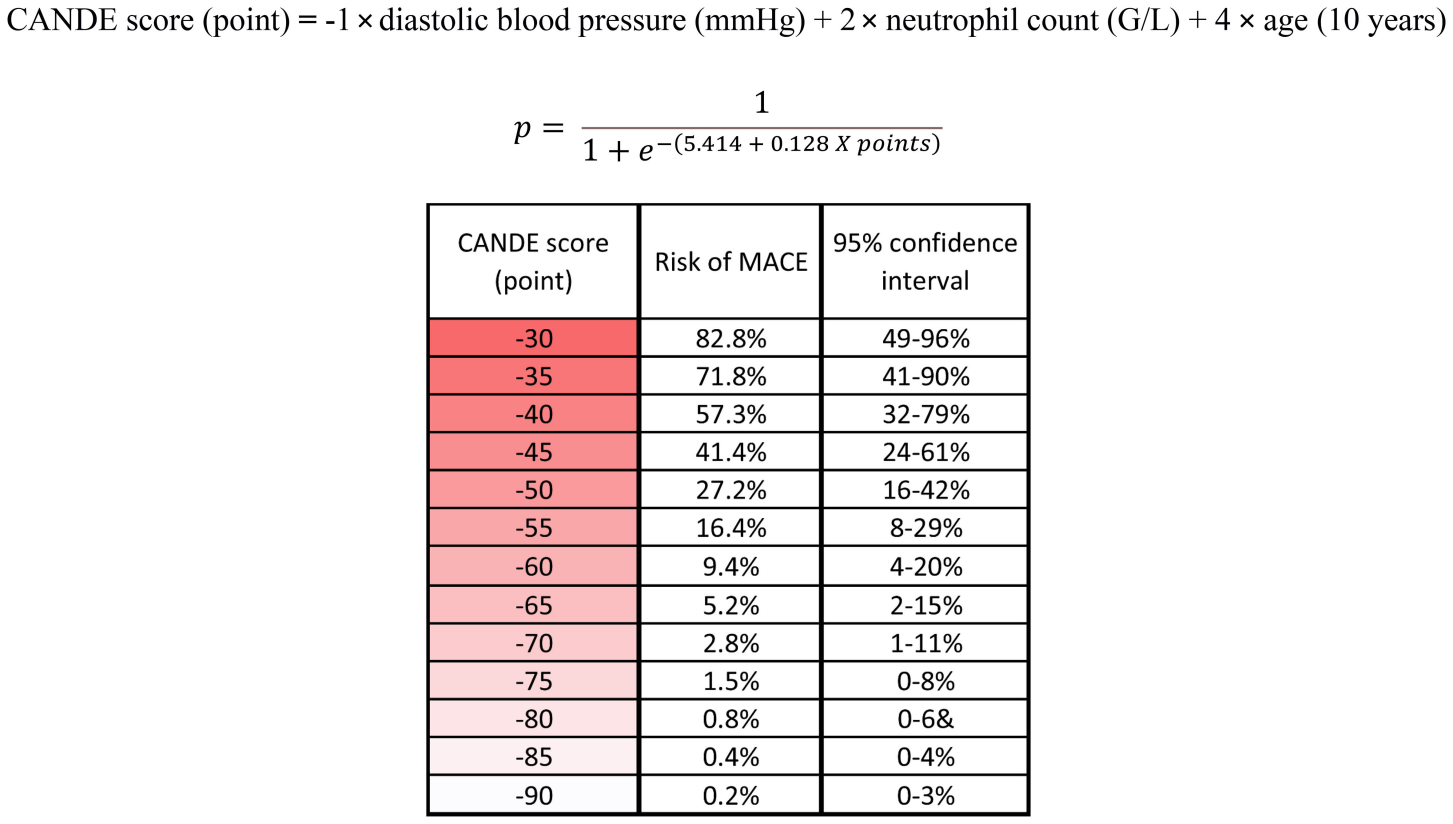
The rounded beta values (coefficients) from the final multivariable logistic regression were used as weighting factors to calculate the CANDE score. The logistic regression model provides a framework for estimating these probabilities to calculate the absolute risk of MACE directly for each patient based on their specific CANDE score (points). The risk assessment table facilitates the practical use of the score. MACE, major adverse cardiovascular event; G/l, Giga/liter; mmHg, Millimeters of Mercury.

The logistic regression model provides a framework for estimating these probabilities to calculate the absolute risk of MACE directly for each patient based on their specific CANDE score (points). By using the intercept (*β_0_*) and the coefficient (*β*), we can transform the individual point scores into probabilities. To facilitate better understanding and practical application, we have formulated a risk assessment table (**Figure 4**, **Supplementary Figure S2**).

**Figure 5:**
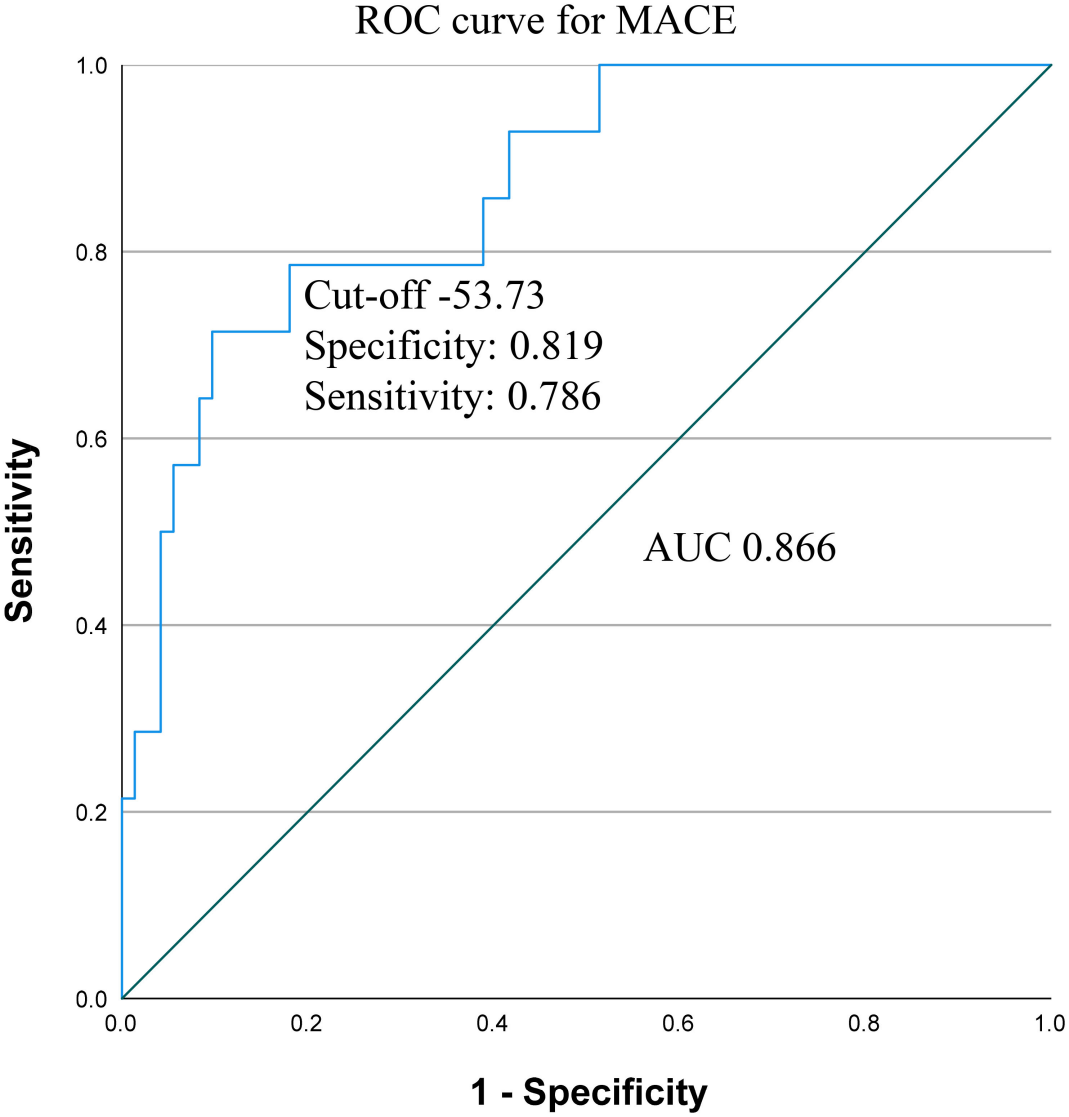
Receiver operating characteristic (ROC) curve and area under the curve (AUC) with cut-off value, sensitivity and specificity for CANDE score predicting MACE. MACE, major adverse cardiovascular event.

CANDE score was applicable either in the group where MACE was examined across the entire medical history (OR 1.137; *p*<0.001), and in the subset where MACE was observed following the renal biopsy (OR 1.081; *p*=0.01) (**Table 4**). The ROC curve analysis found an AUC of 0.866 (95% CI: 0.768-0.965), with a sensitivity of 0.786 and specificity of 0.819 at the optimal cut-off value of -53.73 (**Figure 5**).

## 4 Discussion

Our 16-year retrospective cohort study provides comprehensive insights into CV risk factors associated with lupus nephritis at the time of the biopsy. Lower diastolic blood pressure, higher neutrophil count, and age proved to be independent risk factors for MACEs. Based on a multivariate logistic regression we derived the CANDE score, a tool facilitating the prognostication of MACE in patients diagnosed with lupus nephritis. The CANDE score may become a fast, cheap, and readily available estimation tool at the time of the biopsy to evaluate major adverse cardiovascular risks. This study emphasizes the significance of screening for cardiovascular risks in this particularly susceptible patient population.

While, a conclusion for causality cannot be driven from a prediction model, the main components of our tool are plausibly associated with the risk of CV complications. Diastolic hypotension has been substantiated as an independent risk factor for incident heart failure (7). Whereas cardiovascular risk monotonously increases with higher systolic blood pressure (SBP), the relationship between DBP and cardiovascular diseases has a nonlinear J-shaped association (8). In accordance with findings from NHANES III and the Framingham Heart studies, pulse pressure (PP) tends to rise as DBP decreases during the 6^th^and 7^th^ decades of life. This phenomenon is attributed to arterial stiffness induced by atherosclerosis (9, 10). The stiffening of arteries leads to diminished elastic recoil, decreased arterial compliance, and a subsequent reduction in DBP. Over time, compromised arterial compliance contributes to increased afterload and an elevation in SBP, thereby further expanding the PP, which is associated with adverse cardiovascular and renal outcomes (11). The consequently greater afterload amplifies myocardial oxygen demand, ultimately culminating in myocardial ischemia and the onset of both systolic and diastolic dysfunction (7).

Moreover, DBP governs left coronary perfusion gradient. During diastole, as the myocardium relaxes, the extravascular compression on the left coronaries is alleviated, allowing them to regain complete patency. The patency of the left coronary arteries is contingent upon aortic diastolic pressure. Consequently, coronary perfusion is determined by the difference between the aortic diastolic blood pressure and the left ventricular end-diastolic pressure (12). A decline in DBP reduces coronary blood flow due to the diminished perfusion gradient, leading to myocardial hypoxia and subsequent impairment of contractile function.

These correlations are particularly evident in lupus nephritis patients who exhibit a heightened susceptibility to atherosclerosis, arterial stiffness, coronary artery diseases, and left ventricular hypertrophy as opposed to the general population or individuals with SLE lacking renal involvement (13).

Despite the absence of isolated diastolic hypotension (with DBP ranging from 60-114 mmHg) or significant PP variations within this study, our findings suggest that the marginally lower DBP observed in patients with MACE may indicate an underlying accelerated atherosclerosis, notwithstanding the relatively young age of these individuals and the patient population at large. This finding also emphasizes the need for comprehensive screening and prevention measures in this patient group.

Atherosclerotic plaques are multifaceted formations of vascular and immune cells, manifesting concomitantly with chronic inflammation. Subclinical atherosclerosis is detected in 25-56% of SLE patients, who also exhibit a substantial progression compared to the general population (10% vs 5% per year) (14). Given that immune dysregulation is the hallmark of SLE, it substantively contributes to atherosclerosis. Our study substantiates the pivotal role played by neutrophils in the occurrence of MACEs.

Innate immune dysregulation, with a particular focus on neutrophil granulocytes, significantly contributes to the pathogenesis of cardiovascular complications in SLE patients. Neutrophil granulocytes are the most abundant and rapidly responsive immune cells in the circulation. While their antimicrobial arsenal is robust, it carries several deleterious consequences, including direct organ damage and the release of autoantigens. Of particular interest are low-density granulocytes (LDGs), an abnormal subset of neutrophils in SLE and present as a highly proinflammatory phenotype. Besides the enhanced inflammatory cytokine contribution to accelerated atherosclerosis, LDG cells are more susceptible to spontaneous neutrophil extracellular trap (NET) formation and mitochondrial reactive oxygen species production (15). Overall, NETosis entails an exceptionally high level of inflammatory mediator release, contributing to atherosclerosis.

NETs may also instigate both arterial and venous thrombotic and thromboembolic events. NETs have the potential to provide a scaffold for thrombocyte aggregation and the consolidation of thrombi. Concurrently, local hypoxia induces the release of endothelial procoagulant factors, intensifying the prothrombotic milieu (16). These findings underscore the critical role of innate immune dysregulation, particularly involving neutrophils and NETs, in driving cardiovascular complications in SLE patients.

Sustained glucocorticoid use increases the risk of cardiovascular diseases, contributing to the emergence of major risk factors, including dyslipidemia, obesity, diabetes, and hypertension. Glucocorticoids exert anti-insulin effects, increasing lipolysis and fatty acid release while impeding the uptake and storage of glucose as glycogen while endorsing gluconeogenesis and glycogenolysis. Glucocorticoids stimulate proteolysis, elevating the concentration of amino acids. Additionally, they diminish the translocation of glucose transporters to the cell surface (17). Ultimately, these metabolic alterations culminate in obesity, especially in visceral adiposity, which is recognized as a significant cardiovascular risk factor (18).

Glucocorticoids contribute to impaired vasodilation, increased contractility, and plasma volume expansion, thereby compromising blood pressure regulation and favoring hypertension, leading to cardiac hypertrophy (19).

Thromboembolic complications are more frequent in glucocorticoid administration. Cortisol amplifies procoagulant factors, hematocrit, and viscosity, leading to endothelial dysfunction. These alterations collectively predispose individuals to a state of hypercoagulability (20). Numerous SLE cohort analyses have indicated a correlation between higher cumulative glucocorticoid doses and greater incidence of cardiovascular events (21–24). Our study was not structured to calculate cumulative steroid and other immunosuppressive (ISU) doses. Thus, we posit that this might be one of the reasons why the use of ISUs did not influence MACE occurrence in our study. Moreover, many subjects in our cohort were undergoing low-dose glucocorticoid treatment both at entry and during the maintenance phase, precluding a control group for meaningful comparison.

Anti-dsDNA positivity is recognized as a cardiovascular risk factor in SLE patients, forging connections to increased inflammatory mediators, endothelial dysfunction, and enhanced atherosclerosis. Furthermore, anti-dsDNA positivity is associated with an augmentation of NET-derived molecules such as cell-free nucleosomes, neutrophil elastase, and myeloperoxidase. Thus, anti-dsDNA positivity is linked to a higher cardiovascular risk in general (25, 26). Despite these previous observations, our study revealed an association wherein anti-dsDNA positivity correlated with fewer MACE cases. Moreover, neutrophil count was higher in the anti-dsDNA negative cases. The latter finding correlates with the preceding studies; anti-dsDNA expedited the rate of the apoptosis in neutrophils (27–29). It is important to note that measurement methods varied over the years, and it prevented us from seeking precise correlations in antibody titers, potentially impacting the results. However, the underpinning of our opposing results still necessitates comprehensive elucidation.

Antiphospholipid syndrome (APS) is a significant predisposing factor in atherosclerosis, myocardial infarction, stroke, and valvular heart disease. Antiphospholipid antibodies bind to endothelial β2-glycoprotein1 receptors (β2-GP1) and exert endothelial dysfunction *via* various mechanisms. By inhibiting the endothelial nitric oxide synthase, they counteract several endothelial regulatory mechanisms such as leukocyte adhesion, endothelial cell proliferation, vascular permeability, and smooth muscle cell growth. APS antibodies upregulate leukocyte adhesion molecule expression, concurrently inducing endothelin-1 and tissue factor, thereby promoting thrombocyte aggregation (30). Notably, anti-β2-GP1 and anticardiolipin antibodies mediate the uptake of oxidized low-density lipoproteins by macrophages, underscoring a proatherogenic effect (31, 32).

In addition, valvular involvement emerges as a relatively common cardiac manifestation of APS, occurring in 15-30% of the patients. This involvement typically manifests as valvular thickening, dysfunction, and vegetation, predominantly affecting the atrial aspect of the mitral valve or the vascular surface of the aortic valve. Although the precise pathomechanism remains elusive, it is postulated that anti-β2-GP1 targets the valvular endothelial β2-GP1, eliciting endothelial dysfunction and complement fixation (33).

In conjunction with this evidence, our findings align with the compelling data indicating a higher prevalence of MACE with concurrent APS. This congruence strengthens our current knowledge and highlights the significance of our findings in understanding the broader cardiovascular implications of APS.

Although cardiovascular risk increases vastly in patients with SLE, particularly those with renal involvement, there currently exists no specific guidelines for primary cardiovascular prevention tailored to SLE patients except for APS [ (34–36). General considerations include crucial aspects such as smoking cessation, diabetes mellitus control, and pursuing an active life (37). The administration of statin therapy in SLE patients is advised to adhere to the guidelines established by the American Heart Association and American College of Cardiology’s guidelines (38). Patients maintaining a blood pressure of 130-139/80-89 mmHg or above over a span of two years face a significantly higher chance of atherosclerosis compared to their normotensive counterparts (39). Although there is no specific recommendation for an antihypertensive regimen tailored to lupus patients, angiotensin convertase enzyme inhibitors (ACE-I) are generally used in SLE patients. The LUMINA study suggested that ACE-Is delayed the onset of renal complications and decreased the activity in SLE, potentially serving as a means of primary prevention (40). Furthermore, ACE-I effectively reduces proteinuria, a pivotal cardiovascular risk factor (41). Consequently, the European Alliance of Associations for Rheumatology (EULAR) and European Renal Association (ERA) advocate the implementation of renin-angiotensin-aldosterone blockade, irrespective of lupus nephritis (36, 37, 42). It is noteworthy, however, that in our study only 50.6% of the patients with a confirmed SLE were on ACE-I/ARB at the time of the biopsy, while 9.8% of them with hypertension received no ACE-I/ARB. These findings challenge the alignment of current practices with recommendations and suggest a gap between idealized guidelines and real-world clinical scenarios.

Despite the existence of well-defined risk factors in SLE, the efficacy of primary prevention in this patient cohort is rendered inefficient for several reasons. Given that SLE patients are predominantly of a younger demographic, the conventional risk factors are frequently disregarded and neglected. The lack of testing for established cardiovascular risk factors (e.g., LDL-cholesterol, BMI, diabetes) further highlights the general unawareness within this population. Additionally, there is a shortage of comprehensive literature reviewing specific preventive measures, and guidelines fail to encompass the entirety of the interdisciplinary facets of this disease. This inadequacy is exacerbated by the fact that most landmark trials exclude lupus nephritis patients from the studies. Furthermore, this deficiency is mirrored in the clinical approach, where physicians often gravitate towards addressing monodisciplinary or urgent multidisciplinary concerns, forgetting about long-term, non-specialized issues.

Many established cardiovascular risk assessment tools, such as Framingham Risk Score, SCORE, and Atherosclerotic Cardiovascular Disease (ASCVD) Score, consider predominantly traditional risk elements, neglecting the nuanced impact of a proinflammatory cytokine burden or the extended steroid use. In contrast, the QRISK3 calculator forecasts cardiovascular risk in SLE patients more accurately by incorporating the interplay of both traditional and non-traditional CV risk factors, such as chronic kidney disease and SLE, into consideration (43). The CANDE score is the first lupus nephritis-focused CV risk calculator among these tools. Its unique attribute lies in its applicability at the time of the biopsy, requiring expeditious clinical assessment and an easily accessible laboratory test to prognosticate the potential incidence of major adverse cardiovascular events.

The limitations of our study need to be acknowledged. The analysis confined to a modest sized patient cohort, intrinsically constrained the study’s robustness. While our scrutiny focused on patients undergoing kidney biopsy in our center, a portion of their subsequent care transpired in external institutions, consequently limiting the scope of accessible data. This constraint was further exacerbated by the retrospective nature of our data collection and the single-center focus. Recognizing the impact of race on SLE severity, regrettably, the population of Hungary did not provide an encompassing analysis, it was limited only to Caucasian patients.

Yet, after its external validation, our newly proposed risk assessment score may emerge as an inexpensive and easily accessible tool for patients with lupus nephritis. Hereby we advocate fellow researchers to undertake independent validation studies, to strengthen and enhance the utility of the scoring system in clinical practice.

This study underscores the importance of actively screening for cardiovascular risks within this notably vulnerable patient population. Age, neutrophil count, and diastolic blood pressure have been established as independent risk factors for MACE in lupus nephritis. The CANDE score derived from these parameters, after external validation, may serve as a prompt, cost-effective, and readily accessible estimation tool at the time of the biopsy for assessing the likelihood of major adverse cardiovascular risk.

## Data availability statement

The data analyzed in this study is subject to the following licenses/restrictions: The data presented in this study are available on request from the corresponding author. The data are not publicly available due to privacy reasons. Requests to access these datasets should be directed to NL, ledo.nora@semmelweis.hu.

## Ethics statement

The studies involving humans were approved by Regional and Institutional Committee of Science and Research Ethics of Semmelweis University, Budapest, Hungary. The studies were conducted in accordance with the local legislation and institutional requirements. Written informed consent for participation was not required from the participants or the participants’ legal guardians/next of kin in accordance with the national legislation and institutional requirements.

## Author contributions

AM: Conceptualization, Data curation, Formal analysis, Investigation, Visualization, Writing – original draft. MJ: Investigation, Writing – review & editing. KB: Investigation, Writing – review & editing. AGT: Formal analysis, Writing – review & editing. AT: Resources, Writing – review & editing. NL: Conceptualization, Data curation, Formal analysis, Funding acquisition, Investigation, Resources, Supervision, Writing – review & editing.
