# Supplementary material for: Proposal of a novel cardiovascular risk prediction score in lupus nephritis
Source: Front Immunol. 2024 Jul 24;15:1405463. doi: 10.3389/fimmu.2024.1405463 (PMC11305119; doi:10.3389/fimmu.2024.1405463)
Supplement: Supplementary file 1 [file DataSheet_1.pdf]

# Supplementary Material

## Proposal of a novel cardiovascular risk prediction score in lupus nephritis

Adél Molnár [1], Márk Juha [1], Klaudia Bulajcsík [1], Ádám Gy. Tabák[1,2,3], András Tislér [1] and Nóra Ledó [1,*]

[1] Department of Internal Medicine and Oncology, Semmelweis University, Budapest, Hungary;
Email: molnar.adel@semmelweis.hu (A.M.); juha.mark@semmelweis.hu (M.J.); bulajcsik.klaudia@semmelweis.hu (K.B.); tabak.adam@semmelweis.hu (A.Gy.T.); tisler.andras@semmelweis.hu (A.T.); ledo.nora@semmelweis.hu (N.L.)
[2] Institute of Preventive Medicine and Public Health, Semmelweis University Faculty of Medicine, Budapest, Hungary
[3] UCL Brain Sciences, University College London, London, United Kingdom
***** Correspondence: ledo.nora@semmelweis.hu

**Figure S1. Associations between antiphospholipid syndrome and anticoagulant administration**

All patients with antiphospholipid syndrome (APS) were administered anticoagulants, but not all anticoagulated patients had antiphospholipid syndrome.

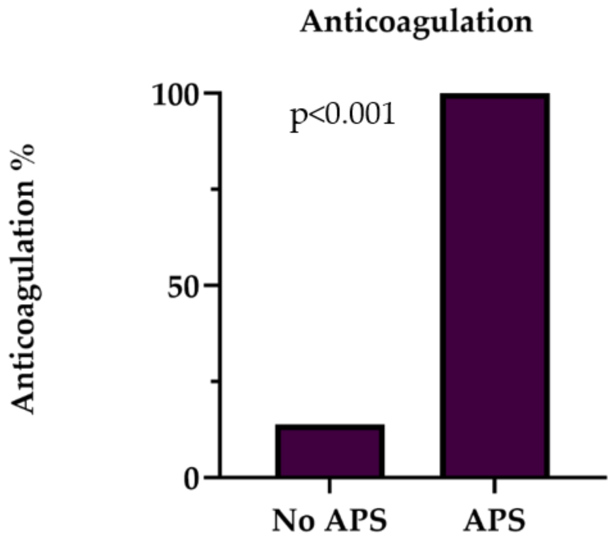

**FigureS2: Development of a risk prediction tool for major adverse cardiovascular events**

**Selection from interrelated variables for the multivariate logistic regression model**

| Interrelated variables | Number of data points | R square from the univariate model |
|---|---|---|
| Absolute neutrophil count | 87 | 0.108 |
| Anti-dsDNA | 74 | 0.105 |
| Anticoagulant use | 91 | 0.155 |
| Deep vein thrombosis | 91 | 0.145 |
| Antiphospholipid syndrome | 91 | 0.123 |

**Multivariate logistic regression – step 1**

| | B | S.E. | Wald | df | Sig. | Exp(B) | 95% C.I. for EXP(B) | |
|---|---|---|---|---|---|---|---|---|
| | | | | | | | Lower | Upper |
| DBP | -.097 | .044 | 4.805 | 1 | .028 | .907 | .832 | .990 |
| NEU | .246 | .121 | 4.118 | 1 | .042 | 1.279 | 1.008 | 1.623 |
| Age | .056 | .029 | 3.762 | 1 | .052 | 1.057 | .999 | 1.119 |
| Beta blocker | .565 | .790 | .511 | 1 | .475 | 1.759 | .374 | 8.274 |
| Anticoagulant | 1.312 | .816 | 2.587 | 1 | .108 | 3.713 | .751 | 18.369 |
| Constant | 2.048 | 3.955 | .268 | 1 | .605 | 7,753 | | |

DBP, diastolic blood pressure (mmHg); NEU, absolute neutrophil count (G/l); Age (years)

**Multivariate logistic regression – step 2**

| | B | S.E. | Wald | df | Sig. | Exp(B) | 95% C.I. for EXP(B) | |
|---|---|---|---|---|---|---|---|---|
| | | | | | | | Lower | Upper |
| DBP | -.099 | .045 | 4.909 | 1 | .027 | .906 | .830 | .989 |
| NEU | .247 | .122 | 4.136 | 1 | .042 | 1.281 | 1.009 | 1.626 |
| Age | .061 | .028 | 4.665 | 1 | .031 | 1.063 | 1.006 | 1.123 |
| Anticoagulant | 1.462 | .782 | 3.493 | 1 | .062 | 4.314 | .931 | 19.979 |
| Constant | 2.079 | 4.013 | .268 | 1 | .604 | 7.995 | | |

DBP, diastolic blood pressure (mmHg); NEU, absolute neutrophil count (G/l); Age (years)

**Multivariate logistic regression – final model**

| | B | S.E. | Wald | df | Sig. | Exp(B) | 95% C.I. for EXP(B) Lower | Upper |
|---|---|---|---|---|---|---|---|---|
| DBP | -0.124 | 0.044 | 7.780 | 1 | 0.005 | 0.884 | 0.810 | 0.964 |
| NEU | 0.278 | 0.119 | 5.401 | 1 | 0.020 | 1.320 | 1.044 | 1.668 |
| Age | 0.052 | 0.026 | 3.899 | 1 | 0.048 | 1.053 | 1.000 | 1.109 |
| Constant | 4.877 | 3.707 | 1.730 | 1 | 0.188 | 131.211 | | |

DBP, diastolic blood pressure (mmHg); NEU, absolute neutrophil count (G/l); Age (years)

$\beta_1$(DBP) = -0.124
$\beta_2$(NEU) = 0.278
$\beta_3$(AGE) = 0.052 → $\beta$(10 years AGE) = 0.52

$$\text{point} = \beta_1 \times \text{diastolic blood pressure} + \beta_2 \times \text{neutrophil count} + \beta_3 \times \text{age}$$

$$\text{point} = -0.124 \times \text{diastolic blood pressure} + 0.278 \times \text{neutrophil count} + 0.52 \times \text{age}$$

**Nearest integer of beta values for easier calculation:**

$\beta_1$(DBP) = -0.124 ~ -1
$\beta_2$(NEU) = 0.278 ~ 2
$\beta_3$(10 years AGE) = 0.52 ~ 4

$$\text{CANDE score (point)} = -1 \times \text{diastolic blood pressure} + 2 \times \text{neutrophil count} + 4 \times \text{age}$$

**Logistic regression with MACE and points**

| | B | S.E. | Wald | df | Sig. | Exp(B) | 95% C.I.for EXP(B) Lower | Upper |
|---|---|---|---|---|---|---|---|---|
| point | 0.128 | 0.035 | 13.519 | 1 | <0.001 | 1.137 | 1.062 | 1.217 |
| Constant | 5.414 | 1.816 | 8.893 | 1 | 0.003 | 224.531 | | |

$$p = \frac{1}{1 + e^{-(\beta 0 + \beta\ X\ points)}}$$

$$p = \frac{1}{1 + e^{-(5.414 + 0.128\ X\ points)}}$$

Absolute risk prediction of MACE:

| CANDE score (point) | Risk of MACE | 95% confidence interval |
|---|---|---|
| -30 | 82.8% | 49-96% |
| -35 | 71.8% | 41-90% |
| -40 | 57.3% | 32-79% |
| -45 | 41.4% | 24-61% |
| -50 | 27.2% | 16-42% |
| -55 | 16.4% | 8-29% |
| -60 | 9.4% | 4-20% |
| -65 | 5.2% | 2-15% |
| -70 | 2.8% | 1-11% |
| -75 | 1.5% | 0-8% |
| -80 | 0.8% | 0-6& |
| -85 | 0.4% | 0-4% |
| -90 | 0.2% | 0-3% |

**Table S1.** Patient characteristics stratified by major adverse cardiovascular event (MACE) status

| Variables | MACE in the medical history[1] | No MACE in the medical history[1] | *p* |
|---|---|---|---|
| Age (years) | 45.50±15.11 (42.50) n=14 | 35.81±11.74 (35.00) n=77 | ***0.012*** |
| Sex (females) | 92.9% (13/14) | 84.4% (65/77) | 0.406 |
| Time from lupus diagnosis to biopsy (years) | 8.80±10.12 (5.50) n=14 | 6.84±6.74 (5.00) n=75 | 0.640 |
| ***Clinical and general laboratory parameters*** | | | |
| Systolic blood pressure (mmHg) | 132.50±18.73 (127.50) n=14 | 141.18±19.92 (140.00) n=74 | 0.069 |
| Diastolic blood pressure (mmHg) | 78.42±6.90 (79.00) n=14 | 89.51±10.96 (90.00) n=74 | ***<0.001*** |
| Pulse pressure (mmHg) | 54.07±19.33 (48.00) n=14 | 51.66±13.61 (52.00) n=74 | 0.842 |
| Weight (kg) | 66.78±9.87 (70.00) n=7 | 66.57±13.72 (65.00) n=47 | 0.632 |
| Height (m) | 150.00±0 (150) n=1 | 166.09±5.77 (168.00) n=11 | 0.167 |
| BMI[2] (kg/m2) | NA[3] | 24.19±3.31 (22.84) n=4 | NA |
| Cholesterol (mmol/l) | 7.18±1.92 (8.10) n=5 | 6.19±2.24 (5.70) n=41 | 0.316 |
| Triglycerides (mmol/l) | 2.49±0.99 (2.69) n=6 | 2.64±1.64 (2.36) n=45 | 0.966 |
| LDL[4] (mmol/l) | 3.97±1.14 (3.61) n=4 | 3.67±1.49 (3.64) n=29 | 0.690 |
| HDL[5] (mmol/l) | 1.97±1.56 (1.63) n=4 | 1.41±0.51 (1.47) n=26 | 0.659 |
| TSH[6] (mU/l) | 6.20±7.56 (3.70) n=5 | 4.10±4.09 (2.66) n=18 | 0.587 |
| D-dimer (mg/l) | 6.02±5.33 (6.02) n=2 | 3.30±3.02 (2.22) n=14 | 0.333 |
| Leukocyte count (G/l) | 9.07±3.25 (8.45) n=14 | 6.99±3.54 (6.50) n=73 | ***0.026*** |
| Hemoglobin (g/l) | 112.21±18.53 (109.00) n=14 | 108.52±18.97 (106.00) n=73 | 0.533 |
| Hematocrit (l/l) | 0.34±0.07 (0.34) n=14 | 0.33±0.06 (0.32) n=73 | 0.595 |
| Neutrophil (%) | 79.14±9.3 (81.25) n=14 | 72.5±12.06 (73.80) n=73 | 0.058 |
| Neutrophil count (G/l) | 7.30±3.11 (6.68) n=14 | 5.15±2.85 (4.65) n=73 | ***0.010*** |
| Lymphocyte (%) | 15.01±8.26 (14.05) n=14 | 19.25±10.06 (17.80) n=73 | 0.146 |
| Lymphocyte count (G/l) | 1.29±0.74 (1.26) n=14 | 1.30±0.98 (1.00) n=73 | 0.599 |
| Platelet count (G/l) | 270.07±100.70 (290.00) n=14 | 245.63±101.25 =242.00) n=73 | 0.212 |
| Sodium (mmol/l) | 139.92±3.25 (140.00) n=13 | 139.85±3.62 (140.00) n=71 | 0.955 |
| Potassium (mmol/l) | 4.37±0.58 (4.30) n=13 | 4.36±0.61 (4.30) n=72 | 0.536 |
| Calcium (mmol/l) | 2.16±0.16 (2.12) n=12 | 2.15±0.22 (2.15) n=63 | 0.745 |
| Phosphate (mmol/l) | 1.38±0.25 (1.35) n=11 | 1.28±0.34 (1.27) n=60 | 0.206 |
| Bilirubin (µmol/l) | 5.83±2.98 (5.30) n=11 | 6.86±3.46 (6.05) n=58 | 0.276 |
| Total serum protein (g/l) | 60.18±11.13 (66.00) n=9 | 59.10±10.48 (59.65) n=60 | 0.521 |
| Serum albumin (g/l) | 30.65±7.78 (27.80) n=11 | 31.19±6.91 (30.75) n=62 | 0.717 |
| CRP[7] (mg/l) | 10.86±13.60 (7.85) n=12 | 7.91±6.91 (3.6) n=62 | 0.304 |
| GFR[8] (ml/min/1.43 m$^2$) | 35.37±14.15 (35.55) n=6 | 47.77±21.88 (49.60) n=33 | 0.184 |
| Creatinine (µmol/l) | 116.15±110.79 (87.00) n=13 | 112.33±89.27 (82.50) n=72 | 0.831 |
| BUN[9] (mmol/l) | 11.98±8.70 (10.30) n=13 | 10.13±7.45 (7.75) n=72 | 0.376 |
| UPCR[10] (mg/mmol) | 759.64±632.14 (583.60) n=9 | 452.37±411.29 (352.45) n=50 | 0.109 |
| UACR[11] (mg/mmol) | 333.44±271.36 (275.15) n=8 | 202.68±216.48 (132.55) n=36 | 0.111 |
| Daily proteinuria (g/day) | 5.61±1.94 (6.06) n=4 | 4.63±3.05 (4.09) n=26 | 0.359 |
| Hematuria (erythrocyte/HPF[12]) | 123.43±366.56 (5.50) n=14 | 27.68±50.95 (10.00) n=71 | 0.648 |
| Leukocyturia (leukocyte/HPF[12]) | 14.29±19.41 (11.50) n=14 | 19.06±46.39 (10.00) n=68 | 0.951 |
| NLR[13] | 7.29±4.99 (5.87) n=14 | 5.68±4.79 (4.18) n=73 | 0.101 |
| NPR[14] | 0.03±0.02 (0.03) n=14 | 0.02±0.02 (0.02) n=73 | 0.111 |
| PLR[15] | 288.40±232.37 (177.88) n=14 | 297.87±315.12 (225.97) n=73 | 0.881 |
| ***Auto-antibodies, lupus-specific laboratory parameters*** | | | |
| C3 (g/l) | 0.69±0.23 (0.67) n=10 | 0.62±0.29 (0.59) n=60 | 0.411 |
| C4 (g/l) | 0.08±0.04 (0.07) n=10 | 0.11±0.11 (0.07) n=60 | 0.880 |
| ANA[16] positivity | 100.0% (13/13) | 93.3% (56/60) | 0.338 |
| Homogenous pattern | 63.6% (7/11) | 54.5% (30/55) | 0.579 |
| Granulated pattern | 36.4% (4/11) | 45.5% (25/55) | 0.743 |

| | | | |
|---|---|---|---|
| Anti-Ribosomal P positivity | 0.0% (0/9) | 5.9% (3/51) | 0.609 |
| Anti-cytoplasmic antibody positivity | 44.4% (4/9) | 32.7% (17/52) | 0.706 |
| Anti-chromatin positivity | 70.0% (7/10) | 72.5% (37/51) | 0.869 |
| Anti-Scl70 positivity | 10.0% (1/10) | 3.8% (2/53) | 0.410 |
| Anti-dsDNA[17] positivity | 63.6% (7/11) | 90.5% (57/63) | ***0.016*** |
| Anti-RNP/Sm positivity | 22.2% (2/9) | 38.0% (19/50) | 0.469 |
| Anti-Smith positivity | 22.2% (2/9) | 26.9% (14/52) | 0.564 |
| Anti-histone positivity | 50.0% (3/6) | 37.8% (14/37) | 0.666 |
| ENA[18] positivity | 85.7% (6/7) | 89.8% (44/49) | 0.744 |
| Anti-SSA (Ro) positivity | 55.6% (5/9) | 64.8% (35/54) | 0.713 |
| Anti-SSB (La) positivity | 0.0% (0/9) | 33.3% (18/54) | ***0.050*** |
| Anti-nucleosome positivity | 83.3% (5/6) | 94.1% (32/34) | 0.394 |
| Anti-C1q positivity | 40.0% (2/5) | 69.6% (32/46) | 0.318 |
| ANCA[19] positivity | 0.0% (0/2) | 19.2% (5/26) | 0.669 |
| Atypical ANCA positivity | 0.0% (0/2) | 25.0% (5/20) | 0.589 |
| pANCA[20] positivity | 0.0% (0/2) | 3.8% (1/26) | 0.929 |
| cANCA[21] positivity | 0.0% (0/2) | 7.7% (2/26) | 0.860 |
| Anticardiolipin positivity | 42.9% (3/7) | 23.9% (11/46) | 0.364 |
| Anti-β2-GPI positivity | 50.0% (3/6) | 22.2% (10/45) | 0.318 |
| Lupus anticoagulant positivity | 66.7% (4/6) | 25.7% (9/35) | 0.069 |
| ***Electrocardiogram parameters*** | | | |
| ST-T deviation | 9.1% (1/11) | 3.5% (2/57) | 0.416 |
| Bundle Branch Block | 10.0/ (1/10) | 3.4% (5/58) | 0.384 |
| ***Comorbidities*** | | | |
| Hypertension | 42.9% (6/14) | 38.2% (29/76) | 0.740 |
| Diabetes mellitus | 14.3% (2/14) | 2.6% (2/76) | 0.113 |
| Deep vein thrombosis | 50.0% (7/14) | 14.3% (11/77) | ***0.002*** |
| Antiphospholipid syndrome | 35.7% (5/14) | 7.8% (6/77) | ***0.011*** |
| Pericardial effusion | 14.3% (2/14) | 11.7% (9/77) | 0.534 |
| Smoking | 33.3/ (4/12) | 27.0% (17/63 | 0.729 |
| ***Medication at the time of the kidney biopsy*** | | | |
| Vitamin D3 | 21.4% (3/14) | 29.9% (23/77) | 0.749 |
| Anticoagulant | 57.1% (8/14) | 19.5% (15/77) | ***0.003*** |
| Thrombocyte aggregation inhibitor | 21.4% (3/14) | 6.5% (5/77) | 0.102 |
| Calcium channel blocker | 28.6% (4/14) | 27.3% (21/77) | 0.575 |
| Spironolactone | 14.3% (2/14) | 2.6% (2/77) | 0.110 |
| Furosemide | 50.0% (7/14) | 32.5% (25/77) | 0.206 |
| Thiazide/thiazide-like diuretics | 21.4% (3/14) | 13.0% (10/77) | 0.683 |
| ACE-I/ARB[22] | 71.4% (10/14) | 44.2% (34/77) | 0.060 |
| Statin | 28.6% (4/14) | 9.1% (7/77) | 0.062 |
| Beta blocker | 50.0% (7/14) | 22.1% (17/77) | ***0.029*** |
| Antimalarial medication | 7.1% (1/14) | 6.8% (5/74) | 0.658 |
| Methotrexate | 0.0% (0/14) | 4.1% (3/74) | 0.591 |
| Mycophenolate-mofetil | 0.0% (0/14) | 4.1% (3/74) | 0.591 |
| Azathioprin | 7.1% (1/14) | 10.8% (8/74) | 0.563 |
| Cyclosporin A | 0.0% (0/14) | 4.1% (3/74) | 0.591 |
| Cyclophosphamide | 0.0% (0/14) | 5.4% (4/74) | 0.611 |

| | | | |
|---|---|---|---|
| Glucocorticoids | 92.9% (13/14) | 81.1% (60/74) | 0.283 |
| ***Remission induction therapy*** | | | |
| EUROLUPUS[23] induction | 54.5% (6/11) | 52.9% (37/70) | 0.917 |
| Cyclophosphamide induction[24] | 0.0% (0/11) | 7.1% (5/70) | 0.606 |
| Glucocorticoids induction only | 0.0% (0/11) | 25.7% (18/70) | 0.111 |
| Plasmapheresis upon induction | 0.0% (0/11) | 4.3% (3/70) | 0.642 |
| Glucocorticoids and calcineurin inhibitor induction | 0.0% (0/11) | 1.4% (1/70) | 0.864 |
| Mycophenolate-mofetil | 0.0% (0/11) | 15.7% (11/70) | 0.345 |
| IVIG[25] upon induction | 0.0% (0/12) | 2.8% (2/72) | 0.733 |
| ***Maintenance therapy*** | | | |
| Methotrexate | 10.0% (1/10) | 1.4% (1/69) | 0.258 |
| Mycophenolate-mofetil | 20.0% (2/10) | 26.1% (18/69) | 0.596 |
| Calcineurin inhibitor maintenance | 20.0% (2/10) | 8.7% (6/69) | 0.591 |
| Antimalarial medication | 20.0% (2/10) | 21.7% (15/69) | 0.873 |
| Azathioprin | 30.0% (3/10) | 31.9% (22/69) | 0.531 |
| Cyclophosphamide | 0.0% (0/10) | 14.5% (10/69) | 0.342 |
| Glucocorticoids | 100.0% (10/10) | 94.2% (65/69) | 0.435 |
| ***Remission – relapses*** | | | |
| Complete remission at 1 year | 75.0% (6/8) | 53.1% (34/64) | 0.240 |
| Partial remission at 1 year | 12.5% (1/8) | 20.3% (13/64) | 0.594 |
| No remission at 1 year | 12.5% (1/8) | 26.6% (17/64) | 0.437 |
| Relapse in 3 years | 66.7% (6/9) | 65.4% (34/52) | 0.940 |
| ***Histopathological data*** | | | |
| Class I | 7.1% (1/14) | 1.3% (1/77) | 0.285 |
| Class II | 7.1% (1/14) | 5.2% (4/77) | 0.575 |
| Class III | 14.3% (2/14) | 23.4% (18/77) | 0.727 |
| Class IV | 50.0% (7/14) | 55.8% (43/77) | 0.774 |
| Class V | 28.6% (4/14) | 19.5% (15/77) | 0.480 |
| Class VI | 0.0% (0/14) | 2.6% (2/77) | 1.000 |
| Overall distribution of the Classes | | | 0.772 |

[1] Data are presented either as % (number/ all patients) or mean ± SD (median) n= number of patients. MACE, major adverse cardiovascular event, [2] BMI, body mass index, [3] NA, not applicable, [4] LDL, low-density lipoprotein, [5] HDL, high-density lipoprotein, [6] TSH, thyroid stimulating hormone, [7] CRP, C-reactive protein, [8] GFR, Glomerular filtration rate, [9] BUN, Blood Urea Nitrogen, [10] UPCR, urinary protein-creatinine ratio, [11] UACR, urinary albumin-creatinine ratio, [12] HPF, high-power field, [13] NLR, Neutrophil-Lymphocyte ratio, [14] NPR, Neutrophil-Platelet ratio, [15] PLR, Platelet-Lymphocyte ratio, [16] ANA, anti-nuclear antibodies, [17] dsDNA, double-stranded deoxyribonucleic acid, [18] ENA, extractable nuclear antigen, [19] ANCA, antineutrophil cytoplasmic antibody, [20] pANCA, perinuclear ANCA, [21] cANCA, cytoplasmic ANCA, [22] ACE-I/ARB, angiotensin-converting enzyme inhibitor/angiotensin II receptor blocker, [23] EUROLUPUS, glucocorticoids and cyclophosphamide, [24] Non-cyclic oral cyclophosphamide, [25] IVIG, intravenous immunoglobulin. Chi-square analysis, Fisher's exact test or Mann-Whitney U-test were utilized to calculate *p*-values. Significant *p*-values are in *italics* and **bold**.

**Table S2. Lower diastolic blood pressure was associated with an increased risk of major adverse cardiovascular events independent of antihypertensive agents**

| Variables | B | *p* | OR[1] | Confidence Interval for OR | |
|---|---|---|---|---|---|
| | | | | Lower | Upper |
| ***Multivariate Logistic Regression*** | | | | | |
| Diastolic blood pressure (mmHg) | -0.109 | ***0.007*** | 0.897 | 0.829 | 0.971 |
| Beta blockers | 1.607 | 0.053 | 4.989 | 0.978 | 25.456 |
| ACE-I/ARB[2] | 1.344 | 0.109 | 3.835 | 0.740 | 19.881 |
| Calcium channel blockers | -1.105 | 0.244 | 0.331 | 0.052 | 2.128 |
| Spironolactone | 1.251 | 0.313 | 3.495 | 0.307 | 39.777 |
| Thiazide/thiazide-like diuretics | -0.797 | 0.402 | 0.451 | 0.070 | 2.911 |
| Furosemide | 0.560 | 0.503 | 1.659 | 0.377 | 7.293 |

[1] OR, odds ratio, [2] ACE-I/ARB, angiotensin-converting enzyme inhibitor, angiotensin receptor blocker. Significant *p*-values are in *italics* and **bold**.

**Table S3. Patient characteristics stratified by coronary revascularization status in the medical history**

| Variables | CR in the medical history[1] | No CR in the medical history[1] | P |
|---|---|---|---|
| Age (years) | 52.33±19.55 (47.00) n=3 | 36.78±11.77 (35.50) n=88 | NS[2] |
| Sex (females) | 100.0% (3/3) | 85.2% (75/88) | NS |
| Time from diagnosing lupus (at the time of the biopsy) (years) | 7.00±6.56 (8.00) n=3 | 7.15±7.40 (5.00) n=86 | NS |
| ***Clinical and general laboratory parameters*** | | | |
| Systolic blood pressure (mmHg) | 134.00±11.53 (135.00) n=3 | 140±20.14 (139.00) n=85 | NS |
| Diastolic blood pressure (mmHg) | 78.33±2.08 (79.00) n=3 | 88.08±11.20 (89.00) n=85 | NS |
| Pulse pressure (mmHg) | 55.67±9.50 (56.00) n=3 | 51.92±14.73 (52.00) n=85 | NS |
| Weight (kg) | 73.67±2.08 (79.00) n=3 | 66.18±13.47 (63.00) n=51 | NS |
| Height (m) | NA[3] | 164.75±7.20 (167.50) n=12 | NA |
| BMI[4] (kg/m2) | NA | 24.19±3.31 (22.84) n=4 | NA |
| Cholesterol (mmol/l) | NA | 6.30±2.21 (5.90) n=46 | NA |
| Triglycerides (mmol/l) | NA | 2.63±1.57 (2.40) n=51 | NA |
| LDL[5] (mmol/l) | NA | 3.71±1.44 (3.64) n=33 | NA |
| HDL[6] (mmol/l) | NA | 1.49±0.72 (1.47) n=30 | NA |
| TSH[7] (mU/l) | NA | 4.56±4.91 (2.67) n=23 | NA |
| D-dimer (mg/l) | NA | 3.64±3.26 (2.54) n=16 | NA |
| Leukocyte count (G/l) | 10.04±1.25 (10.72) n=3 | 7.22±3.58 (6.51) n=84 | NS |
| Hemoglobin (g/l) | 120.00±22.61 (131.00) n=3 | 108.73±18.74 (107.00) n=84 | NS |
| Hematocrit (l/l) | 0.36±0.06 (0.40) n=3 | 0.33±0.06 (0.33) n=84 | NS |
| Neutrophil (%) | 82.13±5.55 (82.30) n=3 | 73.05±11.95 (74.55) n=84 | NS |
| Neutrophil count (G/l) | 8.19±0.68 (8.21) n=3 | 5.40±2.98 (4.81) n=84 | ***0.040*** |
| Lymphocyte (%) | 12.60±3.47 (14.50) n=3 | 18.78±9.97 (17.30) n=84 | NS |
| Lymphocyte count (G/l) | 1.29±0.48 (1.55) n=3 | 1.30±0.96 (1.04) n=84 | NS |
| Platelet count (G/l) | 170.33±105.87 (127.00) n=3 | 252.39±100.31 (251.00) n=84 | NS |
| Sodium (mmol/l) | 138.50±2.12 (138.50) n=2 | 139.89±3.58 (140.00) n=82 | NS |
| Potassium (mmol/l) | 4.35±0.35 (4.35) n=2 | 4.36±0.61 (4.30) n=83 | NS |
| Calcium (mmol/l) | 2.14±0.16 (2.14) n=2 | 2.15±0.21 (2.15) n=73 | NS |
| Phosphate (mmol/l) | 1.22±0.13 (1.22) n=2 | 1.29±0.33 (1.28) n=69 | NS |
| Bilirubin (μmol/l) | 6.10±NA (6.10) n=1 | 6.71±3.41 (5.95) n=68 | NS |
| Total serum protein (g/l) | 68.00±NA (68.00) n=1 | 59.11±10.51 (59.65) n=68 | NS |
| Serum albumin (g/l) | 32.20±7.35 (32.20) n=2 | 31.08±7.03 (30.20) n=71 | NS |
| CRP[8] (mg/l) | 2.78±NA (2.78) n=1 | 8.46±15.25 (3.90) n=73 | NS |

| | | | |
|---|---|---|---|
| GFR[9] (ml/min/1.43 m$^2$) | 51.00±NA (51.00) n=1 | 45.73±21.48 (46.45) n=38 | NS |
| Creatinine (μmol/l) | 78.50±20.51 (78.50) n=2 | 113.75±93.08 (83.00) n=83 | NS |
| BUN[10] (mmol/l) | 11.50±5.09 (11.50) n=2 | 10.39±7.70 (7.80) n=83 | NS |
| UPCR[11] (mg/mmol) | 625.00±295.57 (625.00) n=2 | 494.83±464.56 (399.30) n=57 | NS |
| UACR[12] (mg/mmol) | 293.00±NA (293.00) n=1 | 224.91±232.13 (162.50) n=43 | NS |
| Daily proteinuria (g/day) | 7.38±NA (7.38) n=1 | 4.67±2.93 (4.18) n=29 | NS |
| Hematuria (erythrocyte/HPF[13]) | 1.00±0.00 (1.00) n=3 | 45.00±158.31 (10.00) n=82 | NS |
| Leukocyturia (leukocyte/HPF[13]) | 5.33±6.66 (2.00) n=3 | 18.73±43.66 (11.00) n=79 | NS |
| NLR[14] | 7.06±2.79 (5.61) n=3 | 5.90±4.89 (4.25) n=84 | NS |
| NPR[15] | 0.06±0.03 (0.07) n=3 | 0.02±0.01 (0.02) n=84 | NS |
| PLR[16] | 131.84±53.82 (127.40) n=3 | 297.87±305.89 (131.84) n=84 | NS |
| ***Auto-antibodies, lupus-specific laboratory parameters*** | | | |
| C3 (g/l) | 0.69±0.39 (0.69) n=3 | 0.63±0.28 (0.59) n=68 | NS |
| C4 (g/l) | 0.06±0.02 (0.06) n=2 | 0.11±0.10 (0.07) n=68 | NS |
| ANA[17] positivity | 100.0% (2/2) | 94.4% (67/71) | NS |
| Homogenous pattern | 0.0% (0/1) | 56.9% (37/65) | NS |
| Granulated pattern | 100.0% (1/1) | 43.1% (28/65) | NS |
| Anti-Ribosomal P positivity | 0.0% (0/1) | 5.1% (3/59) | NS |
| Anti-cytoplasmic antibody positivity | 100.0% (1/1) | 33.3% (20/60) | NS |
| Anti-chromatin positivity | 0.0% (0/1) | 73.3% (44/60) | NS |
| Anti-Scl70 positivity | 0.0% (0/1) | 4.8% (3/62) | NS |
| Anti-dsDNA[18] positivity | 100.0% (1/1) | 86.3% (63/73) | NS |
| Anti-RNP/Sm positivity | 0.0% (0/1) | 36.2% (21/58) | NS |
| Anti-Smith positivity | 0.0% (0/1) | 26.7% (16/60) | NS |
| Anti-histone positivity | 100.0% (1/1) | 38.1% (16/42) | NS |
| ENA[19] positivity | 100.0% (1/1) | 89.1% (49/55) | NS |
| Anti-SSA (Ro) positivity | 100.0% (1/1) | 62.9% (39/62) | NS |
| Anti-SSB (La) positivity | 0.0% (0/1) | 29.0% (18/62) | NS |
| Anti-nucleosome positivity | 100.0% (1/1) | 92.3% (36/39) | NS |
| Anti-C1q positivity | NA | NA | NA |
| ANCA[20] positivity | NA | NA | NA |
| Atypical ANCA positivity | NA | NA | NA |
| pANCA[21] positivity | NA | NA | NA |
| cANCA[22] positivity | NA | NA | NA |
| Anticardiolipin positivity | 0.0% (0/1) | 26.9% (14/52) | NS |
| Anti-β2-GPI positivity | 0.0% (0/1) | 26.0% (13/50) | NS |
| Lupus anticoagulant positivity | 100.0% (1/1) | 30.0% (12/40) | NS |
| ***Electrocardiogram parameters*** | | | |
| ST-T deviation | 50.0% (1/2) | 3.0% (2/66) | NS |
| Bundle Branch Block | 0.0% (0/1) | 4.5% (3/67) | NS |
| Pericardial effusion | 0.0% (0/3) | 12.5% (11/88 | NS |
| ***Comorbidities*** | | | |

| | | | |
|---|---|---|---|
| Hypertension | 66.7% (2/3) | 37.9% (33/87) | NS |
| Diabetes mellitus | 33.3% (1/3) | 3.4% (3/87) | NS |
| Deep vein thrombosis | 33.3% (1/3) | 18.4% (16/87) | NS |
| Antiphospholipid syndrome | 33.3% (1/3) | 11.4% (10/88) | NS |
| Smoking | 0.0% (0/3) | 29.2% (21/72) | NS |
| ***Medication at the time of the kidney biopsy*** | | | |
| Vitamin D3 | 0.0% (0/3) | 29.5% (26/88) | NS |
| Anticoagulant | 33.3% (1/3) | 25.0% (22/88) | NS |
| Thrombocyte aggregation inhibitor | 66.7% (2/3) | 6.8% (6/88) | ***0.020*** |
| Calcium channel blocker | 33.3% (1/3) | 27.3% (24/88) | NS |
| Spironolactone | 0.0% (0/3) | 4.5% (4/88) | NS |
| Furosemide | 33.3% (1/3) | 35.2% (31/88) | NS |
| Thiazide/thiazide-like diuretics | 33.3% (1/3) | 13.6% (12/88) | NS |
| ACE-I/ARB[23] | 100.00% (3/3) | 46.6% (41/88) | NS |
| Statin | 33.3% (1/3) | 11.4% (10/88) | NS |
| Beta blocker | 66.7% (2/3) | 25.0% (22/88) | NS |
| Antimalarial medication | 0.0% (0/3) | 7.1% (6/85) | NS |
| Methotrexate | 0.0% (0/3) | 3.5% (3/85) | NS |
| Mycophenolate-mofetil | 0.0% (0/3) | 3.5% (3/85) | NS |
| Azathioprin | 0.0% (0/3) | 10.6% (9/85) | NS |
| Cyclosporin A | 0.0% (0/3) | 3.5% (3/85) | NS |
| Cyclophosphamide | 0.0% (0/3) | 4.7% (4/85) | NS |
| Glucocorticoids | 66.7% (2/3) | 83.5% (71/85) | NS |
| ***Remission induction therapy*** | | | |
| EUROLUPUS[24] induction | 0.0% (0/1) | 53.8% (43/80) | NS |
| Cyclophosphamide induction[25] | 0.0% (0/1) | 6.3% (5/80) | NS |
| Glucocorticoids induction only | 0.0% (0/1) | 22.5% (18/80) | NS |
| Plasmapheresis upon induction | 0.0% (0/1) | 3.8% (3/80) | NS |
| Glucocorticoids and calcineurin inhibitor induction | 0.0% (0/1) | 1.3% (1/80) | NS |
| Mcophenolate-mofetil | 0.0% (0/1) | 13.8% (11/80) | NS |
| IVIG[26] upon induction | 0.0% (0/2) | 2.4% (2/82) | NS |
| ***Maintenance therapy*** | | | |
| Methotrexate | 0.0% (0/1) | 2.6% (2/78) | NS |
| Mycophenolate-mofetil | 0.0% (0/1) | 25.6% (20/78) | NS |
| Calcineurin inhibitor maintenance | 0.0% (0/1) | 10.3% (8/78) | NS |
| Antimalarial medication | 0.0% (0/1) | 21.8% (17/78) | NS |
| Azathioprin | 0.0% (0/1) | 32.1% (25/78) | NS |
| Cyclophosphamide | 0.0% (0/1) | 12.8% (10/78) | NS |
| Glucocorticoids | 100.0% (1/1) | 94.9% (74/78) | NS |
| ***Remission - relapses*** | | | |
| Complete remission at 1 year | 100.0% (2/2) | 54.9% (39/71) | NS |
| Partial remission at 1 year | 0.0% (0/1) | 19.7% (14/71) | NS |

| | | | |
|---|---|---|---|
| No remission at 1 year | 0.0% (0/1) | 25.4% (18/71) | NS |
| Relapse in 3 years | 100.0% (1/1) | 64.4% (38/59) | NS |
| ***Histopathological data*** | | | |
| Class I | 0.0% (0/3) | 2.3% (2/88) | NS |
| Class II | 0.0% (0/3) | 5.7% (5/88) | NS |
| Class III | 33.3% (1/3) | 21.6% (19/88) | NS |
| Class IV | 33.3% (1/3) | 55.7% (49/88) | NS |
| Class V | 33.3% (1/3) | 20.5% (18/88) | NS |
| Class VI | 0.0% (0/3) | 2.3% (2/88) | NS |
| Overall distribution of the Classes | | | NS |

[1] Data are presented either as % (number/ all patients) or mean ± SD (median) n= number of patients. CR, coronary revascularization, [2] NS, not significant, [3] NA, not applicable, [4] BMI, body mass index, [5] LDL, low-density lipoprotein, [6] HDL, high-density lipoprotein, [7] TSH, thyroid stimulating hormone, [8] CRP, C-reactive protein, [9] GFR, Glomerular filtration rate, [10] BUN, Blood Urea Nitrogen, [11] UPCR, urinary protein-creatinine ratio, [12] UACR, urinary albumin-creatinine ratio, [13] HPF, high-power field, [14] NLR, Neutrophil-Lymphocyte ratio, [15] NPR, Neutrophil-Platelet ratio, [16] PLR, Platelet-Lymphocyte ratio, [17] ANA, anti-nuclear antibodies, [18] dsDNA, double-stranded deoxyribonucleic acid, [19] ENA, extractable nuclear antigen, [20] ANCA, antineutrophil cytoplasmic antibody, [21] pANCA, perinuclear ANCA, [22] cANCA, cytoplasmic ANCA, [23] ACE-I/ARB, angiotensin-converting enzyme inhibitor/angiotensin II receptor blocker, [24] EUROLUPUS, glucocorticoids and cyclophosphamide, [25] Non-cyclic oral cyclophosphamide, [26] IVIG, intravenous immunoglobulin. Chi-square analysis, Fisher's exact test or Mann-Whitney U-test were utilized to calculate *p*-values. Significant *p*-values are in *italics* and **bold**.

**Table S4. Patient characteristics stratified by stroke status in the medical history**

| Variables | Stroke in the medical history[1] | No stroke in the medical history[1] | P |
|---|---|---|---|
| Age (years) | 56.20±18.58 (59.00) n=5 | 36.20±10.98 (35.00) n=86 | ***0.017*** |
| Sex (females) | 80.0% (4/5) | 86.0% (74/86) | NS[2] |
| Time from diagnosing lupus (at the time of the biopsy) (years) | 11.42±14.83 (4.00) n=5 | 6.89±6.73 (5.00) n=84 | NS |
| ***Clinical and general laboratory parameters*** | | | |
| Systolic blood pressure (mmHg) | 137.80±19.72 (135.00) n=5 | 139.92±20.02 (139.00) n=83 | NS |
| Diastolic blood pressure (mmHg) | 78.00±4.42 (79.00) n=5 | 88.34±11.18 (89.00) n=83 | ***0.018*** |
| Pulse pressure (mmHg) | 59.08±22.76 (n=56.00) n=5 | 51.58±13.99 (n=52.00) n=83 | NS |
| Weight (kg) | 75.00±NA (75.00) n=1 | 66.44±13.28 (65.00) n=53 | NS |
| Height (m) | NA[3] | 164.75±7.20 (167.50) n=12 | NA |
| BMI[4] (kg/m2) | NA | 24.19±3.31 (22.84) n=4 | NA |
| Cholesterol (mmol/l) | 8.15±0.07 (8.15) n=2 | 6.22±2.22 (5.80) n=44 | NS |
| Triglycerides (mmol/l) | 2.15±1.35 (2.15) n=2 | 2.65±1.59 (2.40) n=49 | NS |
| LDL[5] (mmol/l) | 4.75±1.22 (4.75) n=2 | 3.64±1.44 (3.58) n=31 | NS |
| HDL[6] (mmol/l) | 2.68 ±2.06 (2.68) n=2 | 1.40±0.54 (1.47) n=28 | NS |
| TSH[7] (mU/l) | 8.36±9.83 (5.84) n=3 | 3.99±3.89 (2.66) n=20 | NS |
| D-dimer (mg/l) | NA | 3.64±3.26 (2.54) n=16 | NA |
| Leukocyte count (G/l) | 9.28±2.99 (10.64) n=5 | 7.20±3.57 (6.51) n=82 | NS |
| Hemoglobin (g/l) | 116.60±20.53 (108.00) n=5 | 108.66±18.77 (107.00) n=82 | NS |
| Hematocrit (l/l) | 0.34±0.08 (0.30) n=5 | 0.33±0.06 (0.33) n=82 | NS |
| Neutrophil (%) | 77.90±8.96 (81.20) n=5 | 73.09±12.03 (74.55) n=82 | NS |
| Neutrophil count (G/l) | 7.26±2.64 (6.72) n=5 | 5.39±2.98 (4.81) n=82 | NS |
| Lymphocyte (%) | 117.14±7.87 (14.70) n=5 | 18.65±10.02 (17.30) n=82 | NS |
| Lymphocyte count (G/l) | 1.60±0.98 (1.29) n=5 | 1.28±0.94 (1.01) n=82 | NS |
| Platelet count (G/l) | 254.40±101.30 (295.00) n=5 | 249.27±101.58 (246.50) n=82 | NS |
| Sodium (mmol/l) | 141.00±3.54 (140.00) n=5 | 139.78±3.56 (140.00) n=79 | NS |
| Potassium (mmol/l) | 4.40±0.16 (4.40) n=5 | 4.36±0.62 (4.30) n=80 | NS |
| Calcium (mmol/l) | 2.09±0.15 (2.07) n=4 | 2.16±0.21 (2.15) n=71 | NS |
| Phosphate (mmol/l) | 1.46±0.34 (1.33) n=4 | 1.28±0.33 (1.27) n=67 | NS |
| Bilirubin (µmol/l) | 4.30±0.20 (4.30) n=3 | 6.81±3.43 (6.00) n=66 | NS |
| Total serum protein (g/l) | 53.47±12.81 (54.00) n=3 | 59.50±10.41 (60.65) n=66 | NS |
| Serum albumin (g/l) | 28.23±7.38 (27.40) n=4 | 31.28±6.99 (30.50) n=69 | NS |
| CRP[8] (mg/l) | 2.58±3.27 (1.50) n=4 | 8.72±15.51 (4.15) n=70 | NS |
| GFR[9] (ml/min/1.43 $m^2$) | 35.28±18.24 (39.45) n=4 | 47.07±21.42 (47.70) n=35 | NS |
| Creatinine (µmol/l) | 168.00±166.80 (111.00) n=5 | 109.48±86.12 (81.00) n=80 | NS |
| BUN[10] (mmol/l) | 16.66±11.31 (15.10) n=5 | 10.02±7.26 (7.80) n=80 | NS |
| UPCR[11] (mg/mmol) | 1116.25±803.98 (1042.10) n/4 | 454.37±399.84 (399.30) n=55 | NS |
| UACR[12] (mg/mmol) | 435.07±392.87 (257.30) n=3 | 211.19±213.24 (160.00) n=41 | NS |
| Daily proteinuria (g/day) | 2.95±NA (2.95) n=1 | 4.82±2.95 (4.37) n=29 | NS |
| Hematuria (erythrocyte/HPF[13]) | 15.00±24.11 (5.00) n=5 | 45.23±160.26 (10.00) n=80 | NS |
| Leukocyturia (leukocyte/HPF[13]) | 10.80±5.31 (11.00) n=5 | 18.73±44.26 (10.00) n=77 | NS |
| NLR[14] | 5.33±2.23 (5.61) n=5 | 5.98±4.95 (4.25) n=82 | NS |
| NPR[15] | 0.03±0.02 (0.03) n=5 | 0.02±0.02 (0.02) n=82 | NS |
| PLR[16] | 198.20±127.35 (153.70) n=5 | 297.88±309.12 (212.42) n=82 | NS |

| | | | |
|---|---|---|---|
| ***Auto-antibodies, lupus-specific laboratory parameters*** | | | |
| C3 (g/l) | 0.76±0.34 (0.73) n=4 | 0.62±0.28 (0.59) n=66 | NS |
| C4 (g/l) | 0.09±0.04 (0.09) n=4 | 0.11±0.11 (0.07) n=66 | NS |
| ANA[17] positivity | 100.0% (5/5) | 94.1% (64/68) | NS |
| Homogenous pattern | 75.0% (3/4) | 54.8% (34/62) | NS |
| Granulated pattern | 25.0% (1/4) | 45.2% (28/62) | NS |
| Anti-Ribosomal P positivity | 0.0% (0/3) | 5.3% (3/57) | NS |
| Anti-cytoplasmic antibody positivity | 66.7% (2/3) | 32.8% (19/58) | NS |
| Anti-chromatin positivity | 100.0% (4/4) | 70.2% (40/57) | NS |
| Anti-Scl70 positivity | 0.0% (0/4) | 5.1% (3/59) | NS |
| Anti-dsDNA[18] positivity | 0.0% (0/4) | 91.4% (64/70) | ***<0.001*** |
| Anti-RNP/Sm positivity | 25.0% (1/4) | 36.4% (20/55) | NS |
| Anti-Smith positivity | 25.0% (1/4) | 26.3% (15/57) | NS |
| Anti-histone positivity | 0.0% (0/1) | 40.5% (17/42) | NS |
| ENA[19] positivity | 100.0% (4/4) | 88.5% (46/52) | NS |
| Anti-SSA (Ro) positivity | 75.0% (3/4) | 62.7% (37/59) | NS |
| Anti-SSB (La) positivity | 0.0% (0/4) | 30.5% (18/59) | NS |
| Anti-nucleosome positivity | 50.0% (1/2) | 94.7% (36/38) | NS |
| Anti-C1q positivity | 0.0% (0/2) | 69.4% (34/49) | NS |
| ANCA[20] positivity | NA | NA | NA |
| Atypical ANCA positivity | NA | NA | NA |
| pANCA[21] positivity | NA | NA | NA |
| cANCA[22] positivity | NA | NA | NA |
| Anticardiolipin positivity | 50.0% (1/2) | 25.5% (13/51) | NS |
| Anti-β2-GPI positivity | 50.0% (1/2) | 24.5% (12/49) | NS |
| Lupus anticoagulant positivity | 50.0% (1/2) | 30.8% (12/39) | NS |
| ***Electrocardiogram parameters*** | | | |
| ST-T deviation | 25.0% (1/4) | 3.1% (2/64) | NS |
| Bundle Branch Block | 0.0% (0/3) | 4.6% (3/65) | NS |
| Pericardial effusion | 0.0% (0/5) | 12.8% (11/86) | NS |
| ***Comorbidities*** | | | |
| Hypertension | 60.0% (3/5) | 37.6% (32/85) | NS |
| Diabetes mellitus | 20.0% (1/5) | 3.5% (3/85) | NS |
| Deep vein thrombosis | 20.0% (1/5) | 18.8% (16/85) | NS |
| Antiphospholipid syndrome | 20.0% (1/5) | 11.6% (10.86) | NS |
| Smoking | 20.0% (1/5) | 28.6% (20/70) | NS |
| ***Medication at the time of the kidney biopsy*** | | | |
| Vitamin D3 | 20.0% (1/5) | 29.1% (25/86) | NS |
| Anticoagulant | 40.0% (2/5) | 24.4% (21/86) | NS |
| Thrombocyte aggregation inhibitor | 20.0% (1/5) | 8.1% (7/86) | NS |
| Calcium channel blocker | 40.0% (2/5) | 26.7% (23/86) | NS |
| Spironolactone | 0.0% (0/5) | 4.7% (4/86) | NS |
| Furosemide | 40.0% (2/5) | 34.9% (30/86) | NS |

| | | | |
|---|---|---|---|
| Thiazide/thiazide-like diuretics | 20.0% (1/5) | 14.0% (12/86) | NS |
| ACE-I/ARB[23] | 80.0% (4/5) | 46.5% (40/86) | NS |
| Statin | 60.0% (3/5) | 9.3% (8/86) | ***0.012*** |
| Beta blocker | 60.0% (3/5) | 24.4% (21/86) | NS |
| Antimalarial medication | 20.0% (1/5) | 6.0% (5/83) | NS |
| Methotrexate | 0.0% (0/5) | 3.6% (3/83) | NS |
| Mycophenolate-mofetil | 0.0% (0/5) | 3.6% (3/83) | NS |
| Azathioprin | 20.0% (1/5) | 9.6% (8/83) | NS |
| Cyclosporin A | 0.0% (0/5) | 3.6% (3/83) | NS |
| Cyclophosphamide | 0.0% (0/5) | 4.8% (4/83) | NS |
| Glucocorticoids | 80.0% (4/5) | 83.1% (69/83) | NS |
| ***Remission induction therapy*** | | | |
| EUROLUPUS[24] induction | 75.0% (3/4) | 51.9% (40/77) | NS |
| Cyclophosphamide induction[25] | 0.0% (0/4) | 6.5% (5/77) | NS |
| Glucocorticoids induction only | 0.0% (0/4) | 23.4% (18/77) | NS |
| Plasmapheresis upon induction | 0.0% (0/4) | 3.9% (3/77) | NS |
| Glucocorticoids and calcineurin inhibitor induction | 0.0% (0/4) | 1.3% (1/77) | NS |
| Mycophenolate-mofetil | 0.0% (0/4) | 14.3% (11/77) | NS |
| IVIG[26] upon induction | 0.0% (0/5) | 2.5% (2/79) | NS |
| ***Maintenance therapy*** | | | |
| Methotrexate | 0.0% (0/3) | 2.6% (2/76) | NS |
| Mycophenolate-mofetil | 0.0% (0/3) | 26.3% (20/76) | NS |
| Calcineurin inhibitor maintenance | 9.2% (7/76) | 33.3% (1/3) | NS |
| Antimalarial medication | 33.3% (1/3) | 21.1% (16/76) | NS |
| Azathioprin | 66.7% (2/3) | 30.3% (23/76) | NS |
| Cyclophosphamide | 0.0% (0/3) | 13.2% (10/76) | NS |
| Glucocorticoids | 100.0% (3/3) | 94.7% (72/76) | NS |
| ***Remission - relapses*** | | | |
| Complete remission at 1 year | 100.0% (2/2) | 54.3% (38/70) | NS |
| Partial remission at 1 year | 0.0% (0/2) | 20.0% (14/70) | NS |
| No remission at 1 year | 0.0% (0/2) | 25.7% (18/70) | NS |
| Relapse in 3 years | 50.0% (1/2) | 66.1% (39/59) | NS |
| ***Histopathological data*** | | | |
| Class I | 0.0% (0/5) | 2.3% (2/86) | NS |
| Class II | 0.0% (0/5) | 5.8% (5/86) | NS |
| Class III | 20.0% (1/5) | 22.1% (19/86) | NS |
| Class IV | 40.0% (2/5) | 55.8% (48/86) | NS |
| Class V | 40.0% (2/5) | 19.8% (17/86) | NS |
| Class VI | 0.0% (0/5) | 2.3% (2/86) | NS |
| Overall distribution of the Classes | | | NS |

[1] Data are presented either as % (number/ all patients) or mean ± SD (median) n= number of patients. [2] NS, not significant, [3] NA, not applicable, [4] BMI, body mass index, [5] LDL, low-density lipoprotein, [6] HDL, high-density lipoprotein, [7] TSH, thyroid stimulating hormone, [8] CRP, C-reactive protein, [9] GFR, Glomerular filtration rate, [10] BUN, Blood Urea Nitrogen, [11] UPCR, urinary protein-creatinine ratio, [12] UACR, urinary albumin-creatinine ratio, [13] HPF, high-power field, [14] NLR, Neutrophil-

Lymphocyte ratio, [15] NPR, Neutrophil-Platelet ratio, [16] PLR, Platelet-Lymphocyte ratio, [17] ANA, anti-nuclear antibodies, [18] dsDNA, double-stranded deoxyribonucleic acid, [19] ENA, extractable nuclear antigen, [20] ANCA, antineutrophil cytoplasmic antibody, [21] pANCA, perinuclear ANCA, [22] cANCA, cytoplasmic ANCA, [23] ACE-I/ARB, angiotensin-converting enzyme inhibitor/angiotensin II receptor blocker, [24] EUROLUPUS, glucocorticoids and cyclophosphamide, [25] Non-cyclic oral cyclophosphamide, [26] IVIG, intravenous immunoglobulin. Chi-square analysis, Fisher's exact test or Mann-Whitney U-test were utilized to calculate *p*-values. Significant *p*-values are in *italics* and **bold**.

**Table S5. Patient characteristics by presence or absence of hospitalization due to heart failure**

| Variables | HF in the medical history[1] | No HF in the medical history[1] | P |
|---|---|---|---|
| Age (years) | 40.50±10.33 (42.50) n=6 | 37.07±12.40 (35.00) n=85 | NS[2] |
| Sex (females) | 100.0% (6/6) | 84.7% (72/85) | NS |
| Time from diagnosing lupus (at the time of the biopsy) (years) | 5.21±7.70 (2.00) n=6 | 7.29±7.34 (5.00) n=83 | NS |
| ***Clinical and general laboratory parameters*** | | | |
| Systolic blood pressure (mmHg) | 130.50±21.46 (126.00) n=6 | 140.48±19.74 (139.50) n=82 | NS |
| Diastolic blood pressure (mmHg) | 78.00±10.00 (80.00) n=6 | 88.46±10.95 (89.00) n=82 | ***0.037*** |
| Pulse pressure (mmHg) | 52.5±19.66 (45.50) n=6 | 52.01±14.28 (n=52.00) n=82 | NS |
| Weight (kg) | 64.00±11.14 (62.00) n=3 | 66.75±13.40 (66.00) n=51 | NS |
| Height (m) | 150.00±NA (150.00) n=1 | 166.09±5.77 (168.00) n=11 | NS |
| BMI[3] (kg/m2) | NA[4] | 24.19±3.31 (22.84) n=4 | NA |
| Cholesterol (mmol/l) | 5.20±0.99 (5.20) n=2 | 6.35±2.24 (5.95) n=44 | NS |
| Triglycerides (mmol/l) | 2.30±0.71 (2.50) n=3 | 2.65±1.61 (2.38) n=48 | NS |
| LDL[5] (mmol/l) | 3.21±0.18 (3.21) n=2 | 3.74±1.48 (3.84) n=31 | NS |
| HDL[6] (mmol/l) | 1.28±1.09 (1.28) n=2 | 1.50±0.71 (1.47) n=28 | NS |
| TSH[7] (mU/l) | 2.99±1.02 (2.99) n=2 | 4.71±5.12 (2.67) n=21) | NS |
| D-dimer (mg/l) | 6.03±5.34 (6.03) n=2 | 3.30±3.02 (2.22) n=14 | NS |
| Leukocyte count (G/l) | 9.34±4.08 (7.41) n=6 | 7.17±3.50 (6.70) n=81 | NS |
| Hemoglobin (g/l) | 111.67±18.04 (110.50) n=6 | 108.93±18.99 (106.00) n=81 | NS |
| Hematocrit (l/l) | 0.35±0.07 (0.34) n=6 | 0.33±0.06 (0.32) n=81 | NS |
| Neutrophil (%) | 82.72±6.30 (82.20) n=6 | 72.67±11.93 (74.10) n=81 | NS |
| Neutrophil count (G/l) | 7.87±3.91 (5.92) n=6 | 5.32±2.85 (4.73) n=81 | NS |
| Lymphocyte (%) | 11.37±5.79 (10.65) n=6 | 19.10±9.93 (17.40) n=81 | NS |
| Lymphocyte count (G/l) | 1.02±0.60 (0.93) n=6 | 1.32±0.96 (1.05) n=81 | NS |
| Platelet count (G/l) | 306.00±99.66 (281.00) n=6 | 245.38±100.42 (244.00) n=81 | NS |
| Sodium (mmol/l) | 139.17±3.31 (139.50) n=6 | 139.91±3.58 (140.00) n=78 | NS |
| Potassium (mmol/l) | 4.32±0.85 (4.20) n=6 | 4.36±0.59 (4.30) n=79 | NS |
| Calcium (mmol/l) | 2.20±0.18 (2.16) n=6 | 2.15±0.21 (2.15) n=69 | NS |
| Phosphate (mmol/l) | 1.35±0.23 (1.39) n=5 | 1.29±0.33 (1.28) n=66 | NS |
| Bilirubin (µmol/l) | 7.33±3.02 (5.60) n=6 | 6.64±3.44 (6.00) n=63 | NS |
| Total serum protein (g/l) | 62.64±10.45 (66.60) n=5 | 58.98±10.53 (59.65) n=64 | NS |
| Serum albumin (g/l) | 32.48±8.92 (28.10) n=5 | 31.01±6.90 (30.65) n=68 | NS |
| CRP[8] (mg/l) | 18.13±16.36 (10.80) n=6 | 7.52±14.87 (3.45) n=68 | ***0.021*** |
| GFR[9] (ml/min/1.43 $m^2$) | 34.30±NA (34.30) n=1 | 46.17±21.41 (47.70) n=38 | NS |
| Creatinine (µmol/l) | 76.67±43.81 (64.50) n=6 | 115.67±94.41 (85.00) n=79 | NS |
| BUN[10] (mmol/l) | 9.33±6.60 (8.20) n=6 | 10.49±7.73 (7.90) n=79 | NS |
| UPCR[11] (mg/mmol) | 488.95±335.58 (500.00) n=4 | 499.99±468.79 (399.30) n=55 | NS |
| UACR[12] (mg/mmol) | 267.33±228.51 (234.45) n=4 | 222.37±232.25 (161.25) n=40 | NS |
| Daily proteinuria (g/day) | 6.66±NA (6.66) n=1 | 4.69±2.95 (4.18) n=29 | NS |
| Hematuria (erythrocyte/HPF[13]) | 43.67±56.19 (15.00) n=6 | 43.43±106.92 (9.00) n=79 | NS |
| Leukocyturia (leukocyte/HPF[13]) | 21.33±28.79 (13.00) n=6 | 18.00±43.99 (10.50) n=76 | NS |
| NLR[14] | 9.68±6.43 (8.36) n=6 | 5.66±4.62 (4.24) n=81 | ***0.046*** |
| NPR[15] | 0.03±0.01 (0.02) n=6 | 0.02±0.02 (0.02) n=81 | NS |
| PLR[16] | 427.24±293.33 (379.44) n=6 | 282.14±302.16 (211.94) n=81 | NS |

| | | | |
|---|---|---|---|
| ***Auto-antibodies, lupus-specific laboratory parameters*** | | | |
| C3 (g/l) | 0.71±0.10 (0.78) n=4 | 0.62±0.29 (0.58) n=66 | NS |
| C4 (g/l) | 0.10±0.07 (0.09) n=4 | 0.11±0.10 (0.07) n=66 | NS |
| ANA[17] positivity | 100.0% (6/6) | 94.0% (63/67) | NS |
| Homogenous pattern | 60.0% (3/5) | 55.7% (34/61) | NS |
| Granulated pattern | 40.0% (2/5) | 44.3% (27/61) | NS |
| Anti-Ribosomal P positivity | 0.0% (0/4) | 5.4% (3/56) | NS |
| Anti-cytoplasmic antibody positivity | 25.0% (1/4) | 35.1% (20/57) | NS |
| Anti-chromatin positivity | 50.0% (2/4) | 73.7% (42/57) | NS |
| Anti-Scl70 positivity | 25.0% (1/4) | 3.4% (2/59) | NS |
| Anti-dsDNA[18] positivity | 100.0% (5/5) | 85.5% (59/69) | NS |
| Anti-RNP/Sm positivity | 33.3% (1/3) | 35.7% (20/56) | NS |
| Anti-Smith positivity | 33.3% (1/3) | 25.9% (15/58) | NS |
| Anti-histone positivity | 66.7% (2/3) | 37.5% (15/40) | NS |
| ENA[19] positivity | 50.0% (1/2) | 90.7% (49/54) | NS |
| Anti-SSA (Ro) positivity | 33.3% (1/3) | 65.0% (39/60) | NS |
| Anti-SSB (La) positivity | 0.0% (0/3) | 30.0% (18/60) | NS |
| Anti-nucleosome positivity | 100.0% (2/2) | 92.1% (35/38) | NS |
| Anti-C1q positivity | 66.7% (2/3) | 66.7% (32/48) | NS |
| ANCA[20] positivity | 0.0% (0/2) | 19.2% (5/26) | NS |
| Atypical ANCA positivity | 0.0% (0/2) | 25.0% (5/20) | NS |
| pANCA[21] positivity | 0.0% (0/2) | 3.8% (1/26) | NS |
| cANCA[22] positivity | 0.0% (0/2) | 7.7% (2/26) | NS |
| Anticardiolipin positivity | 66.7% (2/3) | 24.0% (12/50) | NS |
| Anti-β2-GPI positivity | 100.0% (2/2) | 22.4% (11/49) | ***0.061*** |
| Lupus anticoagulant positivity | 50.0% (1/2) | 30.8% (12/39) | NS |
| ***Electrocardiogram parameters*** | | | |
| ST-T deviation | 0.0% (0/5) | 4.8% (3/63) | NS |
| Bundle Branch Block | 20.0% (1/5) | 3.2% (2/63) | NS |
| Pericardial effusion | 33.3% (2/6) | 10.6% (9/85) | NS |
| ***Comorbidities*** | | | |
| Hypertension | 33.3% (2/6) | 39.3% (33/84) | NS |
| Diabetes mellitus | 16.7% (1/6) | 3.6% (3/84) | NS |
| Deep vein thrombosis | 66.7% (4/6) | 15.5% (13/84) | ***0.011*** |
| Antiphospholipid syndrome | 33.3% (2/6) | 10.6% (9/85) | NS |
| Smoking | 75.0% (3/4) | 25.4% (18/71) | ***0.031*** |
| ***Medication at the time of the kidney biopsy*** | | | |
| Vitamin D3 | 33.3% (2/6) | 28.2% (24/85) | NS |
| Anticoagulant | 66.7% (4/6) | 22.4% (19/85) | ***0.034*** |
| Thrombocyte aggregation inhibitor | 0.0% (0/6) | 9.4% (8/85 | NS |
| Calcium channel blocker | 16.7% (1/6) | 28.2% (24/85) | NS |
| Spironolactone | 33.3% (2/6) | 2.4% (2/85) | ***0.021*** |
| Furosemide | 66.7% (4/6) | 32.9% (28/85) | NS |

| | | | |
|---|---|---|---|
| Thiazide/thiazide-like diuretics | 16.7% (1/6) | 14.1% (12/85) | NS |
| ACE-I/ARB[23] | 47.1% (40/85) | 66.7% (4/6) | NS |
| Statin | 0.0% (0/6) | 12.9% (11/85) | NS |
| Beta blocker | 50.0% (3/6) | 24.7% (21/85) | NS |
| Antimalarial medication | 0.0% (0/6) | 7.3% (6/82) | NS |
| Methotrexate | 0.0% (0/6) | 3.7% (3/82) | NS |
| Mycophenolate-mofetil | 0.0% (0/6) | 3.7% (3/82) | NS |
| Azathioprin | 0.0% (0/6) | 11.0% (9/82) | NS |
| Cyclosporin A | 0.0% (0/6) | 3.7% (3/82) | NS |
| Cyclophosphamide | 0.0% (0/6) | 4.9% (4/82) | NS |
| Glucocorticoids | 100.0% (6/6) | 81.7% (67/82) | NS |
| ***Remission induction therapy*** | | | |
| EUROLUPUS[24] induction | 40.0% (2/5) | 53.9% (41/76) | NS |
| Cyclophosphamide induction[25] | 0.0% (0/5) | 6.6% (5/76) | NS |
| Glucocorticoids induction only | 0.0% (0/5) | 23.7% (18/76) | NS |
| Plasmapheresis upon induction | 0.0% (0/5) | 3.9% (3/76) | NS |
| Glucocorticoids and calcineurin inhibitor induction | 0.0% (0/5) | 1.3% (1/76) | NS |
| Mycophenolate-mofetil | 0.0% (0/7) | 14.5% (11/76) | NS |
| IVIG[26] upon induction | 0.0% (0/5) | 2.5% (2/79) | NS |
| ***Maintenance therapy*** | | | |
| Methotrexate | 20.0% (1/5) | 1.4% (1/74) | NS |
| Mycophenolate-mofetil | 40.0% (2/5) | 24.3% (18/74) | NS |
| Calcineurin inhibitor maintenance | 20.0% (1/5) | 9.5% (7/74) | NS |
| Antimalarial medication | 20.0% (1/5) | 21.6% (16/74) | NS |
| Azathioprin | 20.0% (1/5) | 32.4% (24/74) | NS |
| Cyclophosphamide | 0.0% (0/5) | 13.5% (10/74) | NS |
| Glucocorticoids | 100.0% (0/0) | 94.6% (70/74) | NS |
| ***Remission - relapses*** | | | |
| Complete remission at 1 year | 75.0% (3/4) | 54.4% (37/68) | NS |
| Partial remission at 1 year | 25.0% (1/4) | 19.1% (13/68) | NS |
| No remission at 1 year | 0.0% (0/4) | 26.5% (18/68) | NS |
| Relapse in 3 years | 50.0% (2/4) | 66.7% (38/57) | NS |
| ***Histopathological data*** | | | |
| Class I | 16.7% (1/6) | 1.2% (1/85) | NS |
| Class II | 16.7% (1/6) | 4.7% (4/85) | NS |
| Class III | 0.0% (0/6) | 23.5% (20/85) | NS |
| Class IV | 50.0% (3/6) | 55.3% (47/85) | NS |
| Class V | 50.0% (3/6) | 20.0% (17/85) | NS |
| Class VI | 0.0% (0/6) | 2.4% (2/85) | NS |
| Overall distribution of the Classes | | | NS |

[1] Data are presented either as % (number/ all patients) or mean ± SD (median) n= number of patients. HF, hospitalization due to heart failure, [2] NS, not significant, [3] BMI, body mass index, [4] NA, not applicable, [5] LDL, low-density lipoprotein, [6] HDL, high-density lipoprotein, [7] TSH, thyroid stimulating hormone, [8] CRP, C-reactive protein, [9] GFR, Glomerular filtration rate, [10] BUN, Blood Urea Nitrogen, [11] UPCR, urinary protein-creatinine ratio, [12] UACR, urinary albumin-creatinine ratio, [13] HPF, high-power field, [14] NLR, Neutrophil-Lymphocyte ratio, [15] NPR, Neutrophil-Platelet ratio, [16] PLR, Platelet-Lymphocyte ratio, [17]

ANA, anti-nuclear antibodies, [18] dsDNA, double-stranded deoxyribonucleic acid, [19] ENA, extractable nuclear antigen, [20] ANCA, antineutrophil cytoplasmic antibody, [21] pANCA, perinuclear ANCA, [22] cANCA, cytoplasmic ANCA, [23] ACE-I/ARB, angiotensin-converting enzyme inhibitor/angiotensin II receptor blocker, [24] EUROLUPUS, glucocorticoids and cyclophosphamide, [25] Non-cyclic oral cyclophosphamide, [26] IVIG, intravenous immunoglobulin. Chi-square analysis, Fisher's exact test or Mann-Whitney U-test were utilized to calculate *p*-values. Significant *p*-values are in *italics* and **bold**.

**Table S6. Patient characteristics stratified by the presence or absence of acute myocardial infarction**

| Variables | AMI in the medical history[1] | No AMI in the medical history[1] | P |
|---|---|---|---|
| Age (years) | 38.50±12.02 (38.50) n=2 | 37.27±12.32 (36.00) n=89 | NS[2] |
| Sex (females) | 100.0 % (2/2) | 85.4% (76/89) | NS |
| Time from diagnosing lupus (at the time of the biopsy) (years) | 11.00±4.24 (11.00) n=2 | 7.06±7.38 (5.00) n=87 | NS |
| ***Clinical and general laboratory parameters*** | | | |
| Systolic blood pressure (mmHg) | 130.50±20.50 (130.50) n=2 | 140.01±19.95 (139.00) n=86 | NS |
| Diastolic blood pressure (mmHg) | 82.00±2.83 (82.00) n=2 | 87.88±11.24 (88.50) n=86 | NS |
| Pulse pressure (mmHg) | 48.5±23.33 (48.50) n=2 | 52.13±14.50 (52.00) n=86 | NS |
| Weight (kg) | 65.25±15.20 (65.25) n=2 | 66.65±13.29 (65.50) n=52 | NS |
| Height (m) | NA[3] | 164.75±7.20 (167.50) n=12 | NA |
| BMI[4] (kg/m2) | NA | 24.19±3.31 (22.84) n=4 | NA |
| Cholesterol (mmol/l) | 9.20±NA (9.20) n=1 | 6.23±2.19 (5.90) n=50 | NS |
| Triglycerides (mmol/l) | 3.80±NA (3.80) n=1 | 2.60±1.58 (2.38) n=50 | NS |
| LDL[5] (mmol/l) | NA | 3.71±1.44 (3.64) n=33 | NA |
| HDL[6] (mmol/l) | NA | 1.49±0.72 (1.47) n=30 | NA |
| TSH[7] (mU/l) | NA | 4.56±4.91 (2.67) n=23 | NA |
| D-dimer (mg/l) | NA | 3.64±3.26 (2.54) n=16 | NA |
| Leukocyte count (G/l) | 7.96±3.90 (7.96) n=2 | 7.31±3.58 (6.70) n=85 | NS |
| Hemoglobin (g/l) | 93.50±0.71 (93.50) n=2 | 109.48±18.91 (107.00) n=85 | NS |
| Hematocrit (l/l) | 0.29±0.00 (0.29) n=2 | 0.33±0.06 (0.33) n=85 | NS |
| Neutrophil (%) | 67.30±13.01 (67.30) n=2 | 73.50±11.91 (75.00) n=85 | NS |
| Neutrophil count (G/l) | 5.62±3.67 (5.62) n=2 | 5.50±2.99 (4.84) n=85 | NS |
| Lymphocyte (%) | 23.80±13.15 (23.80) n=2 | 18.44±9.86 (17.10) n=85 | NS |
| Lymphocyte count (G/l) | 1.64±0.12 (1.64) n=2 | 1.29±0.95 (1.02) n=85 | NS |
| Platelet count (G/l) | 290.00±1.41 (290.00) n=2 | 248.61±101.98 (245.00) n=85 | NS |
| Sodium (mmol/l) | 139.50±3.54 (139.50) n=2 | 139.87±3.57 (140.00) n=82 | NS |
| Potassium (mmol/l) | 4.45±0.49 (4.46) n=2 | 4.36±0.61 (4.30) n=83 | NS |
| Calcium (mmol/l) | 2.16±0.13 (2.16) n=2 | 2.15±0.21 (2.15) n=73 | NS |
| Phosphate (mmol/l) | 1.29±0.24 (1.29) n=2 | 1.29±0.33 (1.28) n=69 | NS |
| Bilirubin (µmol/l) | 3.60±3.54 (3.60) n=2 | 6.79±3.37 (6.00) n=67 | NS |
| Total serum protein (g/l) | 68.00±NA (68.00) n=1 | 59.11±10.51 (59.65) n=68 | NS |
| Serum albumin (g/l) | 30.95±9.12 (30.95) n=2 | 31.11±7.01 (30.20) n=71 | NS |
| CRP[8] (mg/l) | 5.59±3.97 (5.59) n=2 | 8.46±15.36 (3.80) n=72 | NS |
| GFR[9] (ml/min/1.43 $m^2$) | 36.80±NA (36.80) n=1 | 46.10±21.44 (47.70) n=38 | NS |
| Creatinine (µmol/l) | 105±57.98 (105.00) n=2 | 113.11±93.04 (83.00) n=83 | NS |
| BUN[10] (mmol/l) | 8.25±0.49 (8.25) n=2 | 10.46±7.71 (7.80) n=83 | NS |
| UPCR[11] (mg/mmol) | 416±NA (416.00) n=1 | 500.68±462.61 (400.50) n=758 | NS |
| UACR[12] (mg/mmol) | 293.00±NA (293.00) n=1 | 224.91±232.13 (162.50) n=43 | NS |
| Daily proteinuria (g/day) | 5.47±NA (5.47) n=1 | 4.74±2.97 (4.18) n=29 | NS |
| Hematuria (erythrocyte/HPF[13]) | 695±981.46 (695.00) n=2 | 27.75±49.74 (10.00) n=83 | NS |
| Leukocyturia (leukocyte/HPF[13]) | 2.50±2.12 (2.50) n=2 | 18.64±43.40 (11.00) n=80 | NS |
| NLR[14] | 3.53±2.20 (3.53) n=2 | 6.00±4.86 (4.31) n=85 | NS |
| NPR[15] | 0.02±0.01 (0.02) n=2 | 0.02±0.02 (0.02) n=85 | NS |
| PLR[16] | 177.88±13.94 (177.88) n=2 | 294.84±305.21 (212.90 n=85 | NS |

| | | | |
|---|---|---|---|
| ***Auto-antibodies, lupus-specific laboratory parameters*** | | | |
| C3 (g/l) | 0.46±0.06 (0.46) n=2 | 0.63±0.29 (0.59) n=68 | NS |
| C4 (g/l) | 0.06±0.01 (0.06) n=2 | 0.11±0.10 (0.07) n=68 | NS |
| ANA[17] positivity | 100.0% (2/2) | 94.4% (67/71) | NS |
| Homogenous pattern | 50.0% (1/2) | 56.3% (36/64) | NS |
| Granulated pattern | 50.0% (1/2) | 43.8% (28/64) | NS |
| Anti-Ribosomal P positivity | 0.0% (0/2) | 5.2% (3/58) | NS |
| Anti-cytoplasmic antibody positivity | 50.0% (1/2) | 33.9% (20/59) | NS |
| Anti-chromatin positivity | 50.0% (1/2) | 72.9% (43/59) | NS |
| Anti-Scl70 positivity | 0.0% (0/2) | 4.9% (3/61) | NS |
| Anti-dsDNA[18] positivity | 100.0% (2/2) | 86.1% (62/72) | NS |
| Anti-RNP/Sm positivity | 0.0% (0/2) | 36.8% (21/57) | NS |
| Anti-Smith positivity | 0.0% (0/2) | 27.1% (16/59) | NS |
| Anti-histone positivity | 50.0% (1/2) | 39.0% (16/41) | NS |
| ENA[19] positivity | 100.0% (2/2) | 89.1% (49/55) | NS |
| Anti-SSA (Ro) positivity | 50.0% (1/2) | 63.9% (39/61) | NS |
| Anti-SSB (La) positivity | 0.0% (0/2) | 29.5% (18/61) | NS |
| Anti-nucleosome positivity | 100.0% (2/2) | 92.1% (35/38) | NS |
| Anti-C1q positivity | NA | NA | NA |
| ANCA[20] positivity | NA | NA | NA |
| Atypical ANCA positivity | NA | NA | NA |
| pANCA[21] positivity | NA | NA | NA |
| cANCA[22] positivity | NA | NA | NA |
| Anticardiolipin positivity | 0.0% (0/2) | 27.5% (14/51) | NS |
| Anti-β2-GPI positivity | 0.0% (0/2) | 26.5% (13/49) | NS |
| Lupus anticoagulant positivity | 100.0% (2/2) | 28.2% (11/39) | NS |
| ***Electrocardiogram parameters*** | | | |
| ST-T deviation | 0.0% (0/2) | 4.5% (3/66) | NS |
| Bundle Branch Block | 0.0% (0/2) | 4.5% (3/66) | NS |
| Pericardial effusion | 0.0% (0/2) | 4.5% (3/66) | NS |
| ***Comorbidities*** | | | |
| Hypertension | 50.0% (1/2) | 38.6% (34/88) | NS |
| Diabetes mellitus | 0.0% (0/2) | 4.5% (4/88) | NS |
| Deep vein thrombosis | 100.0% (2/2) | 17.0% (15/88) | ***0.034*** |
| Antiphospholipid syndrome | 100.0% (2/2) | 10.1% (9/89) | ***0.013*** |
| Smoking | 0.0% (0/2) | 28.8% (21/73) | NS |
| ***Medication at the time of the kidney biopsy*** | | | |
| Vitamin D3 | 0.0% (0/2) | 29.2% (26/89) | NS |
| Anticoagulant | 100.0% (2/2) | 23.6% (21/89) | NS |
| Thrombocyte aggregation inhibitor | 100.0% (2/2) | 6.7% (6/89) | ***0.007*** |
| Calcium channel blocker | 50.0% (1/2) | 27.0% (24/89) | NS |
| Spironolactone | 0.0% (0/2) | 4.5% (4/89) | NS |
| Furosemide | 0.0% (0/2) | 36.0% (32/89) | NS |

| | | | |
|---|---|---|---|
| Thiazide/thiazide-like diuretics | 0.0% (0/2) | 14.6% (13/89) | NS |
| ACE-I/ARB[23] | 50.0% (1/2) | 48.3% (43/89) | NS |
| Statin | 50.0% (1/2) | 11.2% (10/89) | NS |
| Beta blocker | 50.0% (1/2) | 25.8% (23/89) | NS |
| Antimalarial medication | 0.0% (0/2) | 7.0% (6/86) | NS |
| Methotrexate | 0.0% (0/2) | 3.5% (3/86) | NS |
| Mycophenolate-mofetil | 0.0% (0/2) | 3.5% (3/68) | NS |
| Azathioprin | 0.0% (0/2) | 10.5% (9/86) | NS |
| Cyclosporin A | 0.0% (0/2) | 3.5% (3/86) | NS |
| Cyclophosphamide | 0.0% (0/2) | 4.7% (4/86) | NS |
| Glucocorticoids | 100.0% (2/2) | 82.6% (71/86) | NS |
| ***Remission induction therapy*** | | | |
| EUROLUPUS[24] induction | 100.0% (1/1) | 52.5% (42/80) | NS |
| Cyclophosphamide induction[25] | 0.0% (0/1) | 6.3% (5/80) | NS |
| Glucocorticoids induction only | 0.0% (0/1) | 22.5% (18/80) | NS |
| Plasmapheresis upon induction | 0.0% (0/1) | 3.8% (3/80) | NS |
| Glucocorticoids and calcineurin inhibitor induction | 0.0% (0/1) | 1.3% (1/80) | NS |
| Mycophenolate-mofetil | 0.0% (0/1) | 138% (11/80) | NS |
| IVIG[26] upon induction | 0.0% (0/1) | 2.4% (2/83) | NS |
| ***Maintenance therapy*** | | | |
| Methotrexate | 0.0% (0/1) | 2.6% (2/78) | NS |
| Mycophenolate-mofetil | 0.0% (0/1) | 25.6% (20/78) | NS |
| Calcineurin inhibitor maintenance | 0.0% (0/1) | 10.30% | NS |
| Antimalarial medication | 0.0% (0/1) | 21.8% (17/78) | NS |
| Azathioprin | 0.0% (0/1) | 32.1 (25/78) | NS |
| Cyclophosphamide | 0.0% (0/1) | 12.8% (10/78) | NS |
| Glucocorticoids | 100% (1/1) | 94.9% (74/78) | NS |
| ***Remission - relapses*** | | | |
| Complete remission at 1 year | 50.0% (1/2) | 55.7% (39/70) | NS |
| Partial remission at 1 year | 0.0% (0/2) | 20.0% (14/70) | NS |
| No remission at 1 year | 50.0% (1/2) | 24.3% (17/70) | NS |
| Relapse in 3 years | 100.0% (2/2) | 64.4% (38/59) | NS |
| ***Histopathological data*** | | | |
| Class I | 0.0% (0/2) | 2.0% (2/89) | NS |
| Class II | 0.0% (0/2) | 5.6% (5/89) | NS |
| Class III | 50.0% (1/2) | 21.2% (19/89) | NS |
| Class IV | 50.0% (1/2) | 55.1% 849/89) | NS |
| Class V | 0.0% (0/2) | 21.3% (19/89) | NS |
| Class VI | 0.0% (0/2) | 2.2% (2/89) | NS |
| Overall distribution of the Classes | | | NS |

[1] Data are presented either as % (number/ all patients) or mean ± SD (median) n= number of patients. AMI, acute myocardial infarct, [2] NS, not significant, [3] NA, not applicable, [4] BMI, body mass index, [5] LDL, low-density lipoprotein, [6] HDL, high-density lipoprotein, [7] TSH, thyroid stimulating hormone, [8] CRP, C-reactive protein, [9] GFR, Glomerular filtration rate, [10] BUN, Blood Urea Nitrogen, [11] UPCR, urinary protein-creatinine ratio, [12] UACR, urinary albumin-creatinine ratio, [13] HPF, high-power field, [14] NLR, Neutrophil-Lymphocyte ratio, [15] NPR, Neutrophil-Platelet ratio, [16] PLR, Platelet-Lymphocyte ratio, [17] ANA, anti-nuclear antibodies, [18] dsDNA, double-stranded deoxyribonucleic acid, [19] ENA, extractable nuclear antigen, [20] ANCA, antineutrophil

cytoplasmic antibody, [21] pANCA, perinuclear ANCA, [22] cANCA, cytoplasmic ANCA, [23] ACE-I/ARB, angiotensin-converting enzyme inhibitor/angiotensin II receptor blocker, [24] EUROLUPUS, glucocorticoids and cyclophosphamide, [25] Non-cyclic oral cyclophosphamide, [26] IVIG, intravenous immunoglobulin. Chi-square analysis, Fisher's exact test or Mann-Whitney U-test were utilized to calculate *p*-values. Significant *p*-values are in *italics* and **bold**.

**Table S7. Patient characteristics stratified by cardiovascular mortality**

| Variables | Cardiovascular death[1] | No cardiovascular death[1] | P |
|---|---|---|---|
| Age (years) | 38.00±0.00 (38.00) n=2 | 36.89±11.88 (36.00) n=87 | NS[2] |
| Sex (females) | 100% (2/2) | 85.1% (74/87) | NS |
| Time from diagnosing lupus (at the time of the biopsy) (years) | 3.50±0.71 (3.50) n=2 | 7.20±7.37 (5.00) n=85 | NS |
| ***Clinical and general laboratory parameters*** | | | |
| Systolic blood pressure (mmHg) | 121.00±4.24 (121.00) n=2 | 140.46±20.08 (139.50) n=84 | NS |
| Diastolic blood pressure (mmHg) | 80.00±5.66 (80.00) n=2 | 88.20±11.19 (89.00) n=84 | NS |
| Pulse pressure (mmHg) | 41.00±1.41 (41.00) n=2 | 52.26±14.80 (52.00) n=84 | NS |
| Weight (kg) | NA[3] | 66.44±13.28 (65.00) n=53 | NA |
| Height (m) | NA | 164.75±7.20 (167.50) n=12 | NA |
| BMI[4] (kg/m2) | NA | 24.19±3.31 (22.84) n=4 | NA |
| Cholesterol (mmol/l) | NA | 6.29±2.23 (5.90) n=45 | NA |
| Triglycerides (mmol/l) | NA | 2.66±1.57 (2.45) n=50 | NA |
| LDL[5] (mmol/l) | NA | 3.71±1.46 (3.61) n=32 | NA |
| HDL[6] (mmol/l) | NA | 1.47±0.73 (1.46) n=29 | NA |
| TSH[7] (mU/l) | 3.71±NA (3.71) n=1 | 4.60±5.02 (2.66) n=22 | NS |
| D-dimer (mg/l) | 2.25±NA (2.25) n=1 | 3.76±3.48 (2.22) n=14 | NS |
| Leukocyte count (G/l) | 5.30±0.98 (5.30) n=2 | 7.36±3.60 (6.70) n=83 | NS |
| Hemoglobin (g/l) | 123±21.21 (123.00) n=2 | 108.81±18.55 (107.00) n=83 | NS |
| Hematocrit (l/l) | 0.37±0.10 (0.37) n=2 | 0.33±0.06 (0.33) n=83.00 | NS |
| Neutrophil (%) | 78.85±3.46 (78.85) n=2 | 73.15±12.09 (74.60) n=83 | NS |
| Neutrophil count (G/l) | 4.21±0.93 (4.21) n=2 | 5.52±3.01 (4.84) n=83 | NS |
| Lymphocyte (%) | 12.05±6.15 (12.05) n=2 | 18.74±10.03 (17.20) n=83 | NS |
| Lymphocyte count (G/l) | 0.61±0.21 (0.61) n=2 | 1.31±0.96 (1.06) n=83 | NS |
| Platelet count (G/l) | 315.00±28.28 (315.00) n=2 | 251.27±100.61 (248.00) n=83 | NS |
| Sodium (mmol/l) | 142.00±1.41 (142.00) n=2 | 139.68±3.43 (140.00) n=80 | NS |
| Potassium (mmol/l) | 4.20±0.28 (4.20) n=2 | 4.36±0.62 (4.30) n=81 | NS |
| Calcium (mmol/l) | 2.24±0.06 (2.24) n=2 | 2.15±0.21 (2.15) n=72 | NS |
| Phosphate (mmol/l) | 1.30±0.13 (1.30) n=2 | 1.29±0.33 (1.28) n=68 | NS |
| Bilirubin (μmol/l) | 4.80±0.71 (4.80) n=2 | 6.76±3.42 (6.00) n=67 | NS |
| Total serum protein (g/l) | 60.55±7.71 (60.55) n=2 | 59.35±10.61 (60.65) n=66 | NS |
| Serum albumin (g/l) | 33.05±7.00 (33.05) n=2 | 30.99±7.04 (30.20) n=69 | NS |
| CRP[8] (mg/l) | 6.50±6.22 (6.50) n=2 | 8.51±15.45 (3.90) n=71 | NS |
| GFR[9] (ml/min/1.43 $m^2$) | 47.70±NA (47.70) n=1 | 46.41±21.61 (46.45) n=36 | NS |
| Creatinine (μmol/l) | 79.00±45.25 (79.00) n=2 | 112.33±92.89 (82.00) n=81 | NS |
| BUN[10] (mmol/l) | 6.55±5.87 (6.55) n=2 | 10.11±7.11 (7.80) n=81 | NS |
| UPCR[11] (mg/mmol) | 416.40±NA (416.40) n=1 | 496.48±468.59 (394.35) n=56 | NS |
| UACR[12] (mg/mmol) | 303.50±NA (303.50) n=1 | 224.67±232.05 (162.50) n=43 | NS |
| Daily proteinuria (g/day) | 2.95±NA (2.95) n=1 | 4.82±2.95 (4.37) n=29 | NS |
| Hematuria (erythrocyte/HPF[13]) | 29.00±41.01 (29.00) n=2 | 44.85±159.29 (10.00) n=81 | NS |
| Leukocyturia (leukocyte/HPF[13]) | 8.00±9.90 (8.00) n=2 | 18.94±43.90 (11.00) n=78 | NS |
| NLR[14] | 7.63±4.18 (7.63) n=2 | 5.93±4.91 (4.26) n=83 | NS |
| NPR[15] | 0.01±0.00 (0.01) n=2 | 0.02±0.02 (0.02) n=83 | NS |
| PLR[16] | 558.21±240.49 (558.21) n=2 | 290.50±303.91 (211.94) n=83 | ***0.045*** |

| | | | |
|---|---|---|---|
| ***Auto-antibodies, lupus-specific laboratory parameters*** | | | |
| C3 (g/l) | 0.80±NA (0.80) n=1 | 0.62±0.28 (0.59) n=68 | NS |
| C4 (g/l) | 0.019±NA (0.019) n=1 | 0.11±0.10 (0.07) n=68 | NS |
| ANA[17] positivity | 100% (2/2) | 94.2% (65/69) | NS |
| Homogenous pattern | 50.0% (1/2) | 55.6% (35/63) | NS |
| Granulated pattern | 50.0% (1/2) | 42.9% (27/63) | NS |
| Anti-Ribosomal P positivity | 0.0% (0/1) | 5.2% (3/58) | NS |
| Anti-cytoplasmic antibody positivity | 0.0% (0/1) | 35.6% (21/59) | NS |
| Anti-chromatin positivity | 100.0% (2/2) | 70.7% (41/58) | NS |
| Anti-Scl70 positivity | 50.0% (1/2) | 3.3% (2/60) | NS |
| Anti-dsDNA[18] positivity | 50.0% (1/2) | 87.3% (62/71) | NS |
| Anti-RNP/Sm positivity | 50.0% (1/2) | 35.7% (20/56) | NS |
| Anti-Smith positivity | 50.0% (1/2) | 25.9% (15/58) | NS |
| Anti-histone positivity | 100.0% (1/1) | 36.6% (15/41) | NS |
| ENA[19] positivity | 100.0% (2/2) | 88.9% (48/54) | NS |
| Anti-SSA (Ro) positivity | 50.0% (1/2) | 65.0% (39/60) | NS |
| Anti-SSB (La) positivity | 0.0% (0/2) | 30.0% (18/60) | NS |
| Anti-nucleosome positivity | 50.0% (1/2) | 94.6% (35/37) | NS |
| Anti-C1q positivity | 100.0% (1/1) | 66.0% (33/50) | NS |
| ANCA[20] positivity | NA | NA | NA |
| Atypical ANCA positivity | NA | NA | NA |
| pANCA[21] positivity | NA | NA | NA |
| cANCA[22] positivity | NA | NA | NA |
| Anticardiolipin positivity | 100.0% (1/1) | 25.5% (13/51) | NS |
| Anti-β2-GPI positivity | 100.0% (1/1) | 24.5% (12/49) | NS |
| Lupus anticoagulant positivity | 100.0% (1/1) | 30.0% (12/40) | NS |
| ***Electrocardiogram parameters*** | | | |
| ST-T deviation | NA | NA | NA |
| Bundle Branch Block | NA | NA | NA |
| Pericardial effusion | 50.0% (1/2) | 11.5% (10/87) | NS |
| ***Comorbidities*** | | | |
| Hypertension | 0.0% (0/2) | 39.5% (34/86) | NS |
| Diabetes mellitus | 0.0% (0/2) | 3.5% (3/86) | NS |
| Deep vein thrombosis | 50.0% (1/2) | 18.6% (16/86) | NS |
| Antiphospholipid syndrome | 0.0% (0/2) | 12.6% (11/87) | NS |
| Smoking | 50.0% (1/2) | 28.2% (20/71) | NS |
| ***Medication at the time of the kidney biopsy*** | | | |
| Vitamin D3 | 0.0% (0/2) | 29.9% (26/87) | NS |
| Anticoagulant | 50.0% (1/2) | 24.1% (21/87) | NS |
| Thrombocyte aggregation inhibitor | 0.0% (0/2) | 8.0% (77/87) | NS |
| Calcium channel blocker | 0.0% (0/2) | 27.6% (24/87) | NS |
| Spironolactone | 0.0% (0/2) | 4.6% (4/87) | NS |
| Furosemide | 100% (2/2) | 33.3% (29/87) | NS |

| | | | |
|---|---|---|---|
| Thiazide/thiazide-like diuretics | 0.0% (0/2) | 14.9% (13/87) | NS |
| ACE-I/ARB[23] | 50.0% (1/2) | 48.3% (42/87) | NS |
| Statin | 50.0% (1/2) | 11.5% (10/87) | NS |
| Beta blocker | 50.0% (1/2) | 24.1% (21/87) | NS |
| Antimalarial medication | 50.0% (1/2) | 6.0% (5/87) | NS |
| Methotrexate | 0.0% (0/2) | 3.6% (3/84) | NS |
| Mycophenolate-mofetil | 0.0% (0/2) | 3.6% (3/84) | NS |
| Azathioprin | 50.0% (1/2) | 9.5% (8/84) | NS |
| Cyclosporin A | 0.0% (0/2) | 3.6% (3/84) | NS |
| Cyclophosphamide | 0.0% (0/2) | 4.8% (4/84) | NS |
| Glucocorticoids | 100% (2/2) | 83.3% (70/84) | NS |
| ***Remission induction therapy*** | | | |
| EUROLUPUS[24] induction | 50.0% (1/2) | 52.6% (41/78) | NS |
| Cyclophosphamide induction[25] | 0.0% (0/2) | 6.4% (5/78) | NS |
| Glucocorticoids induction only | 0.0% (0/2) | 21.8% (17/78) | NS |
| Plasmapheresis upon induction | 0.0% (0/2) | 2.6% (2/78) | NS |
| Glucocorticoids and calcineurin inhibitor induction | 0.0% (0/2) | 1.3% (1/78) | NS |
| Mycophenolate-mofetil | 0.0%(0/2) | 14.1% (11/78) | NS |
| IVIG[26] upon induction | 0.0% (0/2) | 1.3% (1/80) | NS |
| ***Maintenance therapy*** | | | |
| Methotrexate | 0.0% (0/2) | 2.6% (2/76) | NS |
| Mycophenolate-mofetil | 0.0% (0/2) | 26.3% (20/76) | NS |
| Calcineurin inhibitor maintenance | 50.0% (1/2) | 9.2% (7/76) | NS |
| Antimalarial medication | 0.0% (0/2) | 22.4% (17/76) | NS |
| Azathioprin | 50.0% (1/2) | 31.6% (24/76) | NS |
| Cyclophosphamide | 0.0% (0/2) | 13.2% (10/76) | NS |
| Glucocorticoids | 100% (2/2) | 96.1% (73/76) | NS |
| ***Remission - relapses*** | | | |
| Complete remission at 1 year | 100% (2/2) | 55.1% (38/69) | NS |
| Partial remission at 1 year | 0.0% (0/2) | 20.3% (14/69) | NS |
| No remission at 1 year | 0.0% (0/2) | 24.6% (17/69) | NS |
| Relapse in 3 years | 100% (2/2) | 64.4% (38/59) | NS |
| ***Histopathological data*** | | | |
| Class I | 50.0% (1/2) | 1.1% (1/87) | ***0.045*** |
| Class II | 0.0% (0/2) | 5.7% (5/87) | NS |
| Class III | 50.0% (1/2) | 21.8% (19/87) | NS |
| Class IV | 0.0% (0/2) | 56.3% (49/87) | NS |
| Class V | 0.0% 80/2) | 20.7% (18/87) | NS |
| Class VI | 0.0% (0/2) | 2.3% (2/87) | NS |
| Overall distribution of the Classes | | | NS |

[1] Data are presented either as % (number/ all patients) or mean ± SD (median) n= number of patients. [2] NS, not significant, [3] NA, not applicable, [4] BMI, body mass index, [5] LDL, low-density lipoprotein, [6] HDL, high-density lipoprotein, [7] TSH, thyroid stimulating hormone, [8] CRP, C-reactive protein, [9] GFR, Glomerular filtration rate, [10] BUN, Blood Urea Nitrogen, [11] UPCR, urinary protein-creatinine ratio, [12] UACR, urinary albumin-creatinine ratio, [13] HPF, high-power field, [14] NLR, Neutrophil-Lymphocyte ratio, [15] NPR, Neutrophil-Platelet ratio, [16] PLR, Platelet-Lymphocyte ratio, [17] ANA, anti-nuclear antibodies, [18] dsDNA, double-stranded deoxyribonucleic acid, [19] ENA, extractable nuclear antigen, [20] ANCA, antineutrophil cytoplasmic antibody, [21] pANCA, perinuclear

ANCA, [22] cANCA, cytoplasmic ANCA, [23] ACE-I/ARB, angiotensin-converting enzyme inhibitor/angiotensin II receptor blocker, [24] EUROLUPUS, glucocorticoids and cyclophosphamide, [25] Non-cyclic oral cyclophosphamide, [26] IVIG, intravenous immunoglobulin. Chi-square analysis, Fisher's exact test or Mann-Whitney U-test were utilized to calculate *p*-values. Significant *p*-values are in *italics* and **bold**.
